# Clinical and Immunological Metrics During Pediatric Rhesus Macaque Development

**DOI:** 10.3389/fped.2020.00388

**Published:** 2020-07-16

**Authors:** Kristen M. Merino, Nadia Slisarenko, Joshua M. Taylor, Kathrine P. Falkenstein, Margaret H. Gilbert, Rudolf P. Bohm, James L. Blanchard, Amir Ardeshir, Elizabeth S. Didier, Woong-Ki Kim, Marcelo J. Kuroda

**Affiliations:** ^1^Division of Immunology, Tulane National Primate Research Center, Covington, LA, United States; ^2^Walter Reed Army Institute of Research, National Academy of Sciences, Engineering and Medicine Fellow, Silver Spring, MD, United States; ^3^Division of Veterinary Medicine, Tulane National Primate Research Center, Covington, LA, United States; ^4^California National Primate Research Center, School of Veterinary Medicine, University of California, Davis, Davis, CA, United States; ^5^Center for Immunology and Infectious Diseases, California National Primate Research Center, University of California, Davis, Davis, CA, United States; ^6^Division of Microbiology, Tulane National Primate Research Center, Covington, LA, United States; ^7^Department of Microbiology and Molecular Cell Biology, Eastern Virginia Medical School, Norfolk, VA, United States

**Keywords:** infant, hematology, non-human primate, newborn development, nursery

## Abstract

**Background:** Clinical measurements commonly used to evaluate overall health of laboratory animals including complete blood count, serum chemistry, weight, and immunophenotyping, differ with respect to age, development, and environment. This report provides comprehensive clinical and immunological reference ranges for pediatric rhesus macaques over the first year of life.

**Methods:** We collected and analyzed blood samples from 151 healthy rhesus macaques, aged 0–55 weeks, and compared mother-reared infants to two categories of nursery-reared infants; those on an active research protocol and those under derivation for the expanded specific-pathogen-free breeding colony. Hematology was performed on EDTA-anticoagulated blood using a Sysmex XT2000i, and serum clinical chemistry was performed using the Beckman AU480 chemistry analyzer. Immunophenotyping of whole blood was performed with immunofluorescence staining and subsequent flow cytometric analysis on a BD LSRFortessa. Plasma cytokine analysis was performed using a Millipore multiplex Luminex assay.

**Results:** For hematological and chemistry measurements, pediatric reference ranges deviate largely from adults. Comparison of mother-reared and nursery-reared animals revealed that large differences depend on rearing conditions and diet. Significant differences found between two nursery-reared cohorts (research and colony animals) indicate large influences of experimental factors and anesthetic events on these parameters. Immune cells and cytokine responses presented with distinct patterns for infants depending on age, birth location, and rearing conditions.

**Conclusions:** Our results illustrate how the immune system changed over time and that there was variability among pediatric age groups. Reference ranges of results reported here will support interpretations for how infection and treatment may skew common immune correlates used for assessment of pathology or protection in research studies as well as help veterinarians in the clinical care of infant non-human primates. We highlighted the importance of using age-specific reference comparisons for pediatric studies and reiterated the utility of rhesus macaques as a model for human studies. Given the rapid transformation that occurs in multiple tissue compartments after birth and cumulative exposures to antigens as individuals grow, a better understanding of immunological development and how this relates to timing of infection or vaccination will support optimal experimental designs for developing vaccines and treatment interventions.

## Introduction

Immune responses are less developed in pediatric individuals compared to adults for antigen presentation, T cell activation, antibody production, and effector responses (1–4). HIV infections in neonates or infants, for example, result in more rapid and severe disease progression as well as in diminished responsiveness to treatment (5–9). Tobin and Aldrovandi (9) reported that morbidity and mortality rates correlated inversely with age from *in utero* to perinatal and post-natal stages. Interestingly, infected children exhibit variable rates of disease progression and immune responses that appear related to age at infection. For example, a biphasic HIV disease course was observed in infants wherein half progressed rapidly to AIDS and the remainder exhibited persistent infection through adolescence, suggesting that there occurs a transition from pediatric to adult levels of immune responsiveness (10, 11). Thus, the immune system appears to undergo rapid development throughout stages of infancy.

In early life, the immune system is uniquely skewed toward tolerance to avoid development of autoimmune responses and to discriminate commensal organisms from pathogens (12–16). Information currently available about pediatric immunological development mainly derives from studies using the mouse model or human cord blood, both of which have limited translational applications (17–19). For example, clinical parameters in human cord blood, such as neutrophil and lymphocyte numbers, are rarely equivalent to neonatal and infant blood values (20–23). In addition, information in the veterinary medicine literature describes the developmental immune system in companion and food-producing animals but is limited due to stark differences in physiology between these animals and humans.

Rhesus macaques are among the most common non-human primates used in research and closely simulate humans physiologically and immunologically. However, there appears to be relatively little reliable or consistent reference information about immune development of rhesus macaques in the literature. To assess this, a PubMed search was conducted on October 18, 2018 using the following search string: ((((((rhesus OR rhesus monkey OR rhesus macaque OR macaque OR Macaca mulatta)))) AND (((pediatric OR infant OR newborn OR baby OR aging OR development)))) AND (((reference OR reference range^*^ OR normal OR normal values)))) AND (((hematology OR CBC OR lymphocyte^*^ OR granulocyte^*^ OR myeloid OR leukocyte^*^ OR WBC OR white blood cell count))). This search returned 176 results, none of which included comprehensive reference data about rhesus pediatric clinical chemistry or hematology over the course of early development. Historically, studies tended to focus on defining only a few clinical parameters or reported data from relatively wide age ranges among younger animals. Thus, the purpose of this study was to establish the clinical and immunological reference ranges for healthy mother- and nursery-reared rhesus macaques at the Tulane National Primate Research Center (TNPRC) in Covington, LA over the first year of life, accounting for critical details such as housing condition, sex, and maternal status, among others. These results provide a set of reference range values from hematology, blood chemistry, cellular immunophenotyping, and plasma cytokine concentrations. The results further demonstrate the utility of rhesus macaques as a model for studies on human immune system development, and highlight the importance of using age-specific references for pediatric data in research and clinical medicine applications.

## Materials and Methods

### Animals

Rhesus macaques of Indian-ancestry were bred, housed, and evaluated at the TNPRC in accordance with the Animal Welfare Act, the Guide for the Care, and Use of Laboratory Animals (24) and other federal statutes and regulations relating to experiments involving animals. The TNPRC is AAALAC accredited, and all protocols and procedures were reviewed and approved by the Tulane University Institutional Animal Care and Use Committee. Animals received routine veterinary care and specimens were collected from June 2017 through July 2018. For this study, 151 animals ranging from 0.00 to 1.08 years of age (71 females, 80 males) were sampled (Table S1). Among these were mother-reared (MR) as well as nursery-reared (NR) infants housed in the general animal breeding colony or assigned to research studies.

From the mother-reared infants in the breeding colony (Colony MR), 127 samples were collected from 117 infants ranging from 0.00 to 1.00 years of age that were raised by their birth or foster dam. The cohort was evenly divided by sex, with 65 samples from females and 62 samples from males. The infants typically were considered nutritionally weaned at 4–6 months of age, but may have continued to nurse for comfort beyond that time. Most of these infants (112/117) were born in the TNPRC specific pathogen-free (SPF) breeding colony, which is free of *Macacine herpesvirus* 1 (formerly *Cercopithecine herpesevirus*), Simian Immunodeficiency Virus, Simian Betaretrovirus, and Simian T-Cell Lymphotropic Virus. The remaining five infants were born to females in the expanded SPF (eSPF) breeding colony, which also is free of simian foamy virus, rhesus cytomegalovirus, rhesus rhadinovirus, simian virus 40, lymphocryptovirus, measles virus and simian varicella virus. Eight of the 127 samples were collected from seven animals after tetanus vaccination and were 0.71–1.01 years of age at the time of sampling and 18–113 days after vaccination. Most samples (113/127) were collected from animals that had lived in large outdoor social groups prior to sample collection, but all were housed indoors at the time of sample collection. Two animals were delivered by Caesarian section and examined by a veterinarian prior to sample collection. The remaining animals were born via unassisted vaginal deliveries. Since there were limited numbers of samples collected from breeding-colony mother-reared infants within the first 2 weeks of life, the age-binning data only, was supplemented with additional data from specimens obtained from 3 mother-reared infants which had hematology and clinical chemistry data available at 0–2 weeks of age. These 3 infants (1 female, 2 males) were born via unassisted vaginal delivery and were mother-reared indoors for a separate study. No experimental manipulations beyond routine clinical care were performed on these infants or their dams. Retrospective analysis of animal records identified four samples (2 females, 2 males) that were collected from animals not meeting the study's definition of “healthy” and those samples were excluded from analysis, resulting in a total of 123 samples from 114 unique animals for analysis.

An additional 53 samples were collected from 21 nursery-reared colony (Colony NR) infants at 0.11–1.03 years of age that were born into the SPF breeding colony via unassisted vaginal delivery and transferred to a dedicated nursery before 1 week of age (range 1–5 days old). This cohort was evenly divided by sex, with 25 samples collected from females and 28 samples collected from males. From this group, 31 samples were collected from 13 animals born outdoors and 22 samples were from eight animals born indoors. Following maternal separation, neonates were placed in an incubator for 24–48 h, transferred to standard nursery caging, and then placed in continuous social housing beginning at 14 days of age. Infants were fed a standard formula (Similac Advance OptiGRO, Abbott Nutrition, Columbus, OH, USA) initially via hand bottle feeding and transitioned bottle-stand feeding at seven days of age. By 28 days of age, infants accessed free-bottle feeding and began receiving cereal mash, biscuits softened in formula, and fruit or fruit puree. Infants began transitioning from formula to solid biscuits by 120 days of age.

Additional nursery-reared infants that were assigned to a pediatric SIV infection research study (Research NR) were sampled here as well and comprised. This group consisted of 10 infants (6 females, 4 males). These animals were born via unassisted vaginal delivery and transferred to a dedicated nursery before 1 week of age (range 1–7 days). Seven animals in this cohort were born outdoors. All animals in this cohort were peer-reared in the nursery with feeding and husbandry routines as described for the Colony NR cohort. These infants were sampled biweekly from birth until ~5 months of age or until just prior to inoculation with SIV. Two uninfected control animals (1 female, 1 male) continued biweekly sample collections until ~1 year of age. In addition to routine clinical care, animals in this cohort underwent several research interventions, including regular anesthetic events, administration of non-infectious reagents such as dextran and 5-bromo-2′-deoxyuridine (BrdU), and surgical biopsies.

The primary differences between mother-reared and nursery-reared groups were diet and environmental exposures. Both nursery-reared cohorts underwent maternal separation at a similar time post-birth, and were reared under similar conditions (i.e., formula-fed, housed indoors and peer-reared beginning at 2–4 weeks of age). Differences between Research NR and Colony NR groups included anesthesia and sampling frequency, research procedures (e.g., BrdU and dextran injections), as well as biopsy collections. Colony NR animals did not undergo research procedures, and anesthetic events were limited to those needed for clinical care and routine preventive and medicine screenings. It is important to note that due to study design limitations the majority of Colony MR animals were sampled once, while NR groups were samples longitudinally, with Research NR animals sampled the most frequently.

### Venipuncture, Hematology, and Blood Chemistry

Animals were anesthetized with ketamine hydrochloride (10 mg/kg intramuscularly) for femoral venipuncture. From younger infants, samples were collected by gentle aspiration using a needle and syringe, transferred to sample tubes (Sarstedt S-Monovette 06.1667.001 and Sarstedt Multivette 15.1671.100), and inverted to mix gently. From larger infants, blood was collected directly into the sample tubes and inverted to mix. Hematology was performed on EDTA-anticoagulated blood using a Sysmex XT2000i and blood chemistry was performed using the Beckman AU480 chemistry analyzer.

### Flow Cytometry

Whole blood aliquots were stained with two optimized panels of antibodies followed by incubation with RBC lysis buffer (BD Biosciences). The first panel comprised monoclonal antibodies (mAbs) CXCR3-Alexa Fluor (IC6) 488, CCR5-PE (3A9), CD28-PE-CF594 (28.2), CCR6-PE-Cy7 (11A9), CD95-APC (DX2), CD3-AF700 (SP34-2), CD4-APC-H7 (L200), and CD8-V500 (SK1) for staining and 8-color flow cytometric analysis. The second panel included mAbs CD20-PE-CF594 (2H7), CD123-PerCP-Cy5.5 (7G3), HLA-DR-PE-Cy7 (L243), CD1c-APC (BDCA-1), CD11b-AF700 (ICRF44), CD16-APC-H7 (3G8), CD14- Pacific Blue (PB) (M5E2), CD3-V500 (SP34-2), and CD8-Brilliant Violet (BV) 650 (SK1) for staining and 9-color flow cytometric analysis. All mAbs utilized in this study were obtained from either BD Biosciences, Biolegend, or Miltenyi Biotec. Cells were surface stained and resuspended in PBS containing 2% paraformaldehyde, and results were acquired on an LSR Fortessa. Data were analyzed using FlowJo software (TreeStar) and immunophenotyping characterizations were as detailed in Table S2.

### Cytokine Quantification

Infant plasma was prepared by centrifugation of whole blood at 500 × *g* for 10 min and stored in aliquots at −80 degrees Celsius. For analyses, plasma samples were thawed, filtered to remove debris and assayed for multiplex cytokine quantification per manufacturer instructions (Milliplex Non-Human Primate Cytokine Magnetic Bead Panel—Premixed 23 Plex, Cat #PCYTMG-40K-PX23; EMD Millipore Corporation, Billerica, MA).

### Statistical Analysis

The non-parametric Mann-Whitney test was used to compare results between two unmatched treatment groups and Kruskal-Wallis test was used for comparing results between three or more unmatched groups coupled with Dunn's multiple-comparison *post-hoc* test. Linear regression analysis was applied for comparing slopes of results by ANCOVA. GraphPad Prism 7.0 software for Mac (San Diego, CA USA; www.graphpad.com) was used to analyze data and prepare figures. Statistical significance was considered as *P* < 0.05.

## Results

### Weights in Growing Infants

**Figure 1:**
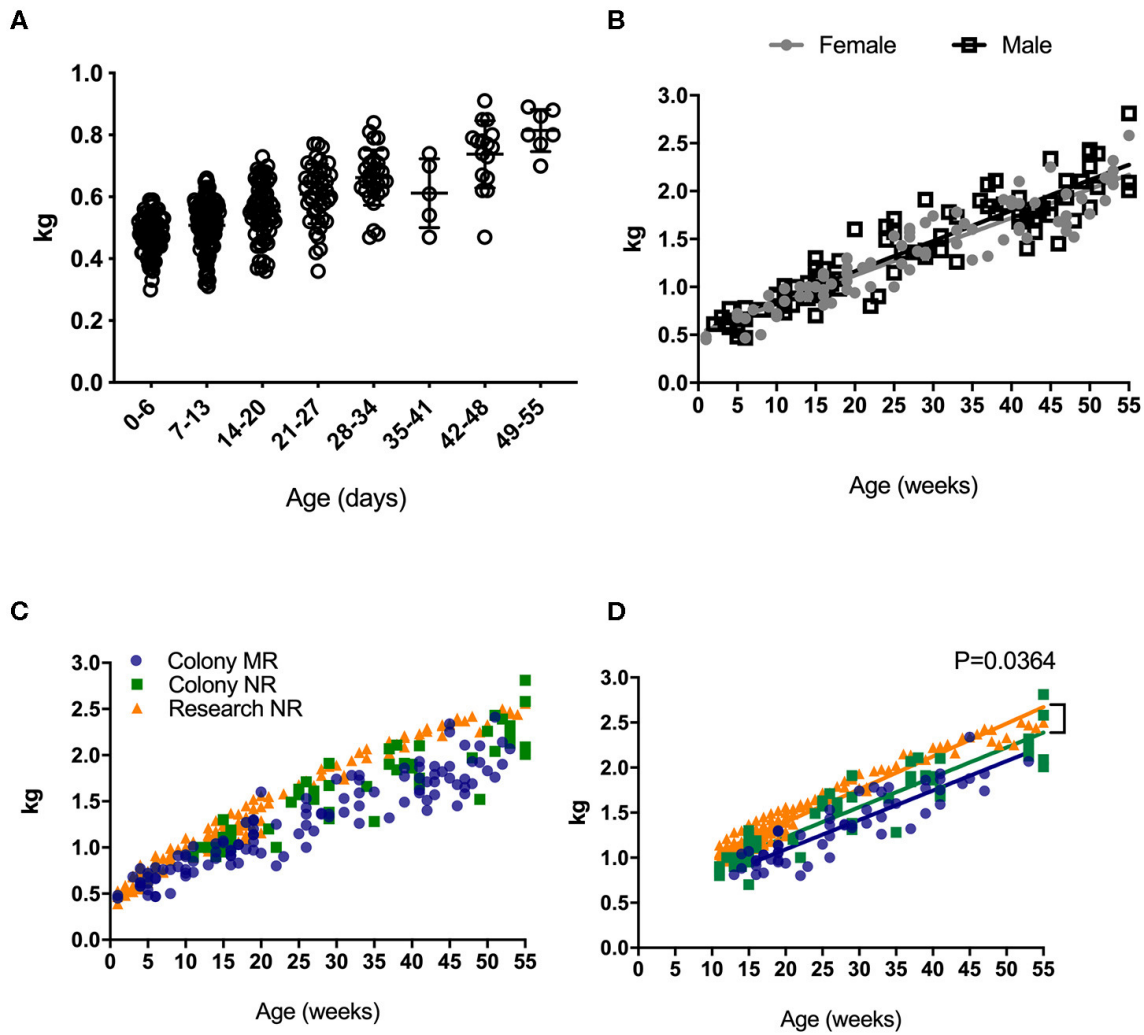
Weights of pediatric rhesus macaques. Weights were plotted over the first 55 days for all pediatric groups including Colony MR and Colony NR, as well as Research NR animals **(A)**. Weights were stratified by sex in colony animals through ~1 yr of age **(B)**. Weights were plotted for groups of Colony MR (blue circles), Colony NR (green squares) and Research NR (orange triangles) animals **(C)**. Linear regression of weights were plotted in each group of animals from Panel C after normalizing for age and slopes were compared by ANCOVA; *P* < 0.05 was considered significant **(D)**.

Weight is universally used to assess health and normal development, and increased rapidly in rhesus macaques after birth (Figure 1A). An early plateau or loss in weight over time may indicate failure to thrive that can quickly translate to a clinical endpoint requiring euthanasia in non-human primates under clinical or research investigations. Thus, reference growth charts specific to rearing and study conditions for infant rhesus macaques are important for guiding such decisions. There were no statistically significant differences in the rates of growth between female and male infants (Figure 1B). In addition to comparing results from all samples collected (for all ages collected) (Figure 1C), the data were normalized for sampling age (Figure 1D), to control for time in all groups where slope was being compared. In both comparisons, no significantly different rates of growth between mother-reared (breast-fed) and nursery-reared (formula-fed) animals (Figures 1C,D). There were, however, significant differences in rates of weight gain between Research NR animals and Colony NR animals (*P* = 0.0364), despite identical diets and rearing conditions (Figure 1D).

### Pediatric Clinical Reference Ranges

Results from complete blood counts (CBC) and blood chemistry analyses from normal, healthy colony animals (both MR and NR) as well as research (NR) animals are summarized in Table 1. The reference ranges were proposed based on means ± 2 Std. Dev. values generated from our pediatric colony and research cohorts aged 0–55 weeks. Individual animal results were plotted for comparison to standard adult reference ranges used by the TNPRC clinical pathology lab. Data were further stratified by specific rearing condition to describe proposed pediatric reference ranges (mean, St. Dev or SD, 95% CI, median, range, and N), and provided in Tables S3A,B. Regression analyses were subsequently performed for comparing slopes along with age binning comparisons to help further extract important divergences between pediatric cohorts.

**Table 1:** Hematology and blood chemistry values of pediatric colony and research rhesus macaques.

**Parameter**	Proposed Range (colony)^*^	Proposed Range (research)^*^	**Units**
% Neutrophils	7.9–63.2	5.0–55.0	
% Lymphocytes	31.0–84.9	39.2–86.9	
% Monocytes	1.3–6.6	1.4–9.2	
% Eosinophils	0–8.0	0–3.6	
% Basophils	0–1.1	0–1.1	
# Neutrophils	0–7.67	0–4.89	X 10^3^/uL
# Lymphocytes	0.26–11.07	1.63–7.70	X 10^3^/uL
# Monocytes	0–0.82	0.09–0.67	X 10^3^/uL
# Eosinophils	0–1.15	0–0.28	X 10^3^/uL
# Basophils	0–0.12	0–0.08	X 10^3^/uL
WBC	1.59–18.47	3.30–11.64	X 10^3^/uL
RBC	4.72–6.70	4.55–6.20	X 10^6^/uL
Hemoglobin	9.4–14.9	10.2–14.9	g/dL
Hematocrit	31.4–45.1	33.1–45.3	%
MCV	54.3–80.4	63.9–82.4	fL
MCH	16.3–26.4	19.9–26.9	pg
MCHC	29.1–34.2	30.3–33.6	g/dL
RDW	8.1–21.5	11.7–14.2	%
Platelets	164.2–798.5	218.5–731.0	X 10^3^/uL
MPV	9.6–13.6	9.7–14.1	fL
Sodium	139–149	141–148	mEq/L
Potassium	2.2–6.3	2.7–6.2	mEq/L
Chloride	103–113	105–113	mEq/L
Total Protein	5.2–7.3	4.9–6.9	g/dL
Albumin	3.4–4.7	3.1–4.5	g/dL
Globulin	1.4–3.0	1.4–2.7	g/dL
Calcium	9.1–11.1	9.1–10.9	mg/dL
Total Bilirubin	0–0.39	0–0.33	mg/dL
BUN	5.2–26.5	0.6–25.2	mg/dL
Glucose	20–145	21–131	mg/dL
Creatinine	0.15–0.58	0.19–0.37	mg/dL
Phosphorus	5.4–9.3	6.3–10.6	mg/dL
Iron	8–228	51.0–263.8	ug/dL
Alkaline Phosphatase	239–1,368	393–1,498	mg/dL
AST	0–181	0–142	U/L
ALT	2–54	11–62	U/L
LDH	0–1,746	0–1,682	U/L
Cholesterol	70–248	94–182	mg/dL
Triglycerides	12–102	0–130	mg/dL
GGT	28–153	42–138	U/L

* *ranges were based on ± 2 standard deviations from the mean and 0 was used if the lower range value was <0*.

#### Hematology

**Figure 2:**
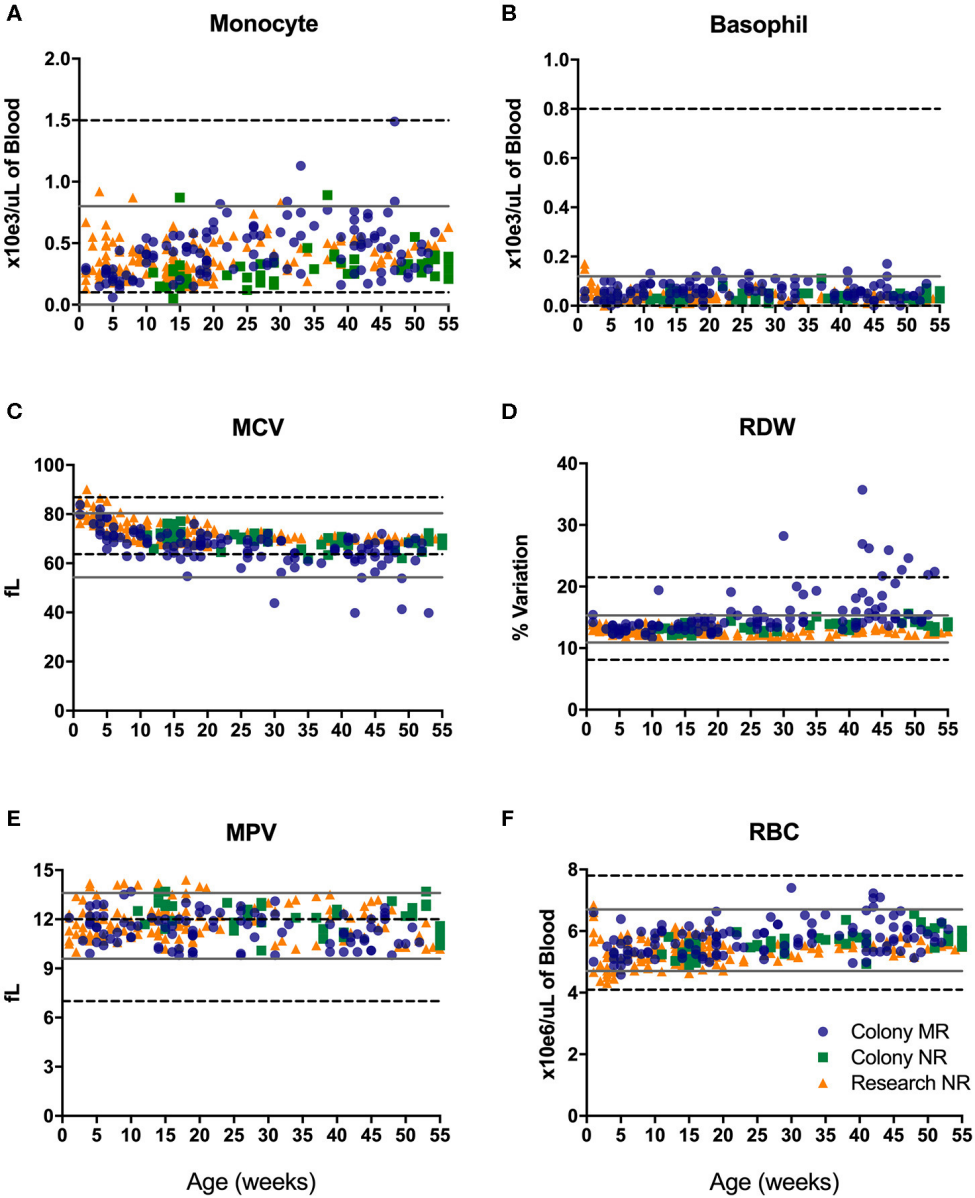
Reference ranges of selected CBC results from pediatric infant animals (gray solid lines plotted as +/– 2 std. dev. of the mean) illustrate differences from established adult rhesus macaque reference ranges (dotted black lines). Individual data points were from animals in the Colony MR (blue circles), Colony NR (green squares), and Research NR (orange triangles) groups comparing; monocyte number **(A)**, basophil number **(B)**, mean corpuscular volume or MCV **(C)**, red cell distribution width or RDW **(D)**, mean platelet volume or MPV **(E)**, and red blood cell count or RBC **(F)**.

CBC values provide information related to hematopoiesis, growth, metabolism, and immune status. The proposed reference ranges for infants differed from the adult rhesus macaque in-house ranges for numbers of monocytes (Figure 2A) and basophils (Figure 2B), as well as in mean corpuscular volume (MCV; Figure 2C), red cell distribution width (RDW; Figure 2D), mean platelet volume (MPV; Figure 2E) and red blood cell numbers (RBC; Figure 2F). Also apparent were differences between previously established adult ranges compared to proposed pediatric reference ranges for white blood cell count (WBC) and % hematocrit (HCT) (not shown). These results demonstrate differences in CBC and blood chemistry value ranges between adult and pediatric rhesus macaques.

**Figure 3:**
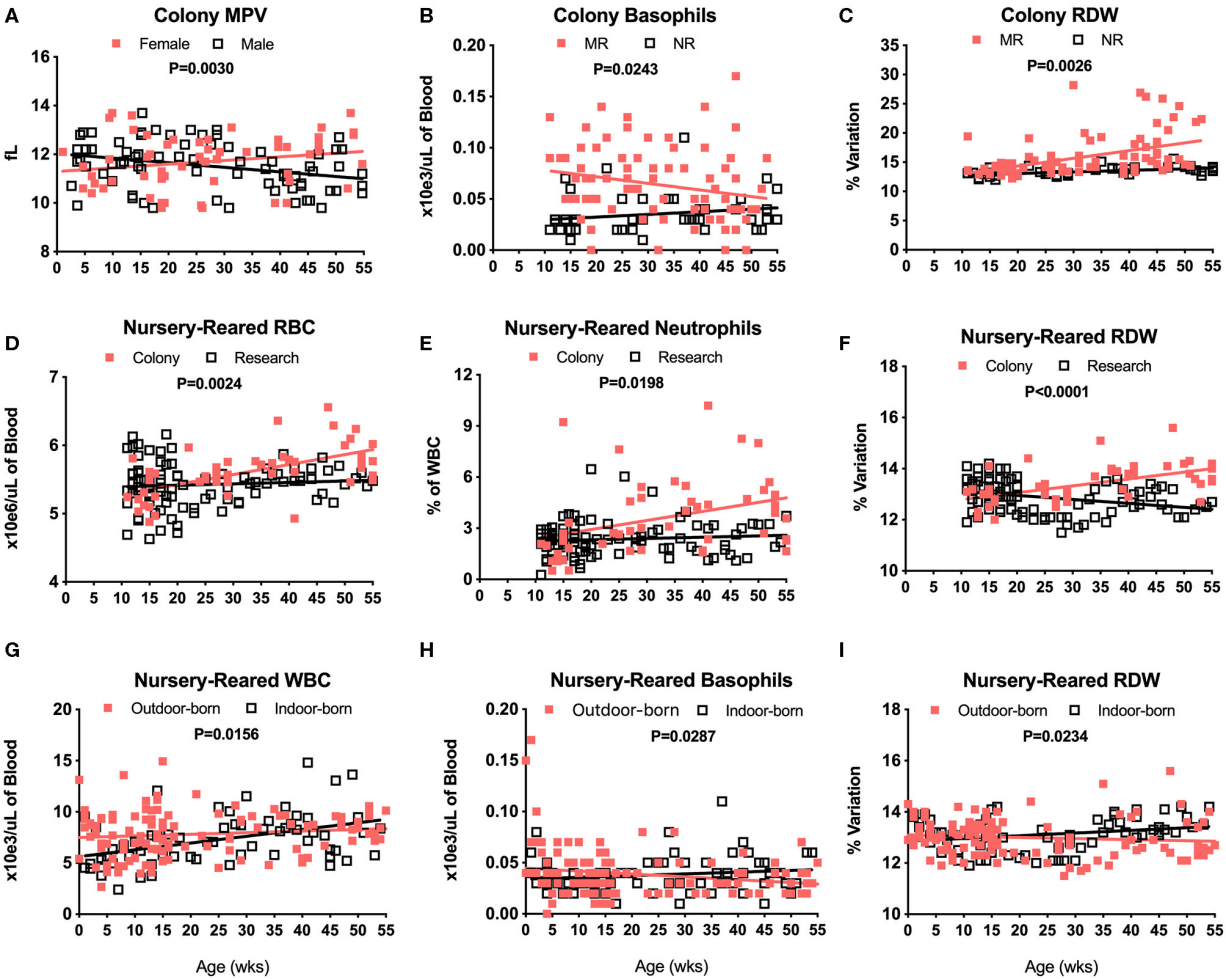
Relationships were assessed between selected CBC values and groups of colony and nursery-reared pediatric animals over age at time of specimen collection. Linear regression and ANCOVA were performed to compare slopes between male and female colony animal MPV data for increasing age (Male: slope = −0.0188, *R*^2^ = 0.1016; Female: slope = 0.015, *R*^2^ = 0.0434) **(A)** and in Colony MR and Colony NR animals over age for number of basophils (MR: slope = 0.0006, *R*^2^ = 0.0518; NR: slope = 0.0003, *R*^2^ = 0.0382) **(B)** and RDW (MR: slope = 0.0282, *R*^2^ = 0.2117; NR: slope = 0.0269, *R*^2^ = 0.2931) **(C)**. Linear regression comparisons via ANCOVA between Colony NR and Research NR animals over age were plotted for RBC count (Colony: slope = 0.0146, *R*^2^ = 0.3454; Research: slope = 0.0021, *R*^2^ = 0.0072) **(D)**, neutrophil numbers (Colony: slope = 0.0531, *R*^2^ = 0.1205; Research: slope = 0.0076, *R*^2^ = 0.0092) **(E)**, and RDW (Colony: slope = 0.0269, *R*^2^ = 0.2931; Research: slope = −0.0156, *R*^2^ = 0.0992) **(F)**. Linear regressions and ANCOVA comparing NR animals born outdoors vs. indoors were shown for WBC numbers (Outdoor: slope = 0.0156, *R*^2^ = 0.0144; Indoor: slope = 0.0661, *R*^2^ = 0.1888) **(G)**, basophil numbers (Outdoor: slope = −0.0003, *R*^2^ = 0.0384; Indoor: slope = 0.0002, *R*^2^ = 0.0233) **(H)**, and RDW (Outdoor: slope = −0.0043, *R*^2^ = 0.0099; Indoor: slope = 0.0105, *R*^2^ = 0.0681) **(I)**.

There were no significant differences in CBC values between male and female pediatric macaques with the exception of MPV (Figure 3A, *P* = 0.0030). We did, however, detect significant differences between Colony MR and Colony NR animals in numbers of basophils (Figure 3B, *P* = 0.0243), RDW (Figure 3C, *P* = 0.0026) and HCT (data not shown, *P* = 0.0110). Among the nursery-reared cohorts, significant differences also were observed between colony and research animals in RBC counts (Figure 3D, *P* = 0.0024), numbers of neutrophils (Figure 3E, *P* = 0.0198), and RDW (Figure 3F, *P* < 0.0001) as well as for HCT (*P* = 0.0035), MCHC (*P* = 0.0173), WBC numbers (*P* = 0.0320), and HGB (*P* = 0.0007) (data not shown). Differences between indoor and outdoor birth locations of nursery-reared animals also were measured in numbers of WBC (Figure 3G, *P* = 0.0156) and basophils (Figure 3H, *P* = 0.0287), as well as RDW (Figure 3I, *P* = 0.0234), and MCV (data not shown, *P* = 0.0422), indicating that these parameters may be affected by perinatal environmental factors.

**Figure 4:**
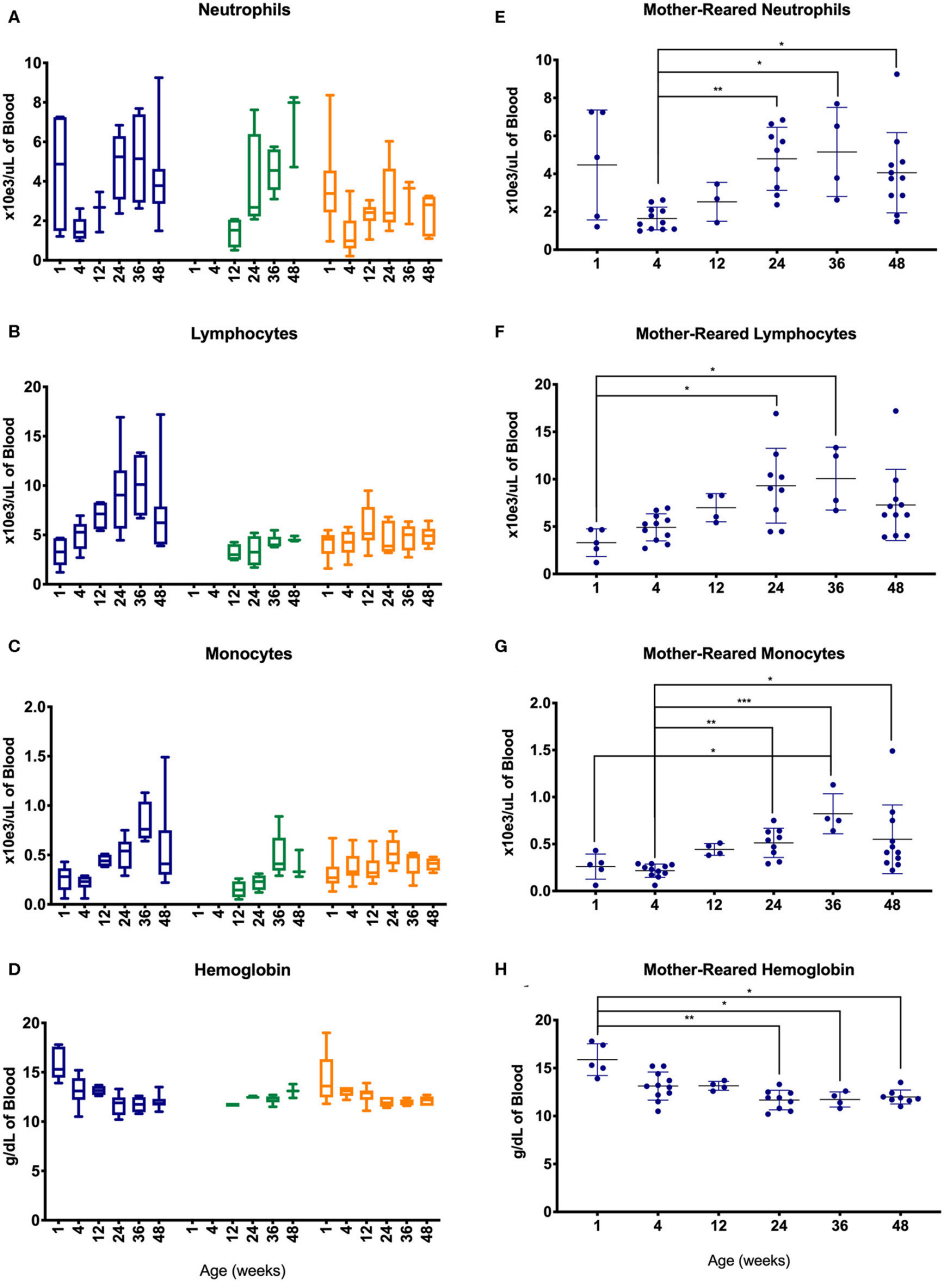
Comparison of selected CBC parameters among pediatric groups of animals over time. Age binning was completed for samples from animals with average ages of 1 week (0–2 weeks old), 4 weeks (3–5 weeks old), 12 weeks (11–13 weeks old), 24 weeks (22–26 weeks old), 36 weeks (34–38 weeks old), and 48 weeks (46–50 weeks old). Data were shown for neutrophil numbers **(A)**, lymphocyte numbers **(B)**, monocyte numbers **(C)**, and hemoglobin **(D)** in animals from the Colony MR (blue), Colony NR (green), and Research NR (orange). **(A–D)** were plotted using box and whiskers depicting median, minimum and maximum values and **(E–H)** applied statistical analyses of results (mean ± st. dev.) from the Colony MR animals using Kruskal-Wallis test coupled with Dunn's multiple comparisons to illustrate how CBC parameters vary with age. *P < 0.05; **P < 0.01; ***P < 0.001.

To further evaluate changes in CBC values, specimens were assessed between different age groups of the pediatric animals—i.e., after binning samples within defined age groups (Figure 4). Graphs shown in Figures 4A–D illustrate effects of rearing conditions on CBC results from Colony MR, Colony NR, and Research NR animals associated with rearing. Colony MR and Colony NR animals shared similar patterns in neutrophil numbers over time (Figure 4A), while the two nursery-reared cohorts were most similar for numbers of lymphocyte numbers and shifts over time (Figure 4B). For monocyte numbers (Figure 4C), values increased with age in the colony animals, but remained relatively stable in research animals. For Research MR and Research NR groups, the neutrophil counts observed at ~1 week of age differed vastly from the remaining age groups within the cohorts (Figures 4A,D).

Not all of the pediatric groups of animals were sampled within the first 4 weeks of life, and the highest numbers of samples evaluated were from the Colony MR. Thus, non-parametric one-way ANOVA was performed to compare age-related differences within this Colony MR cohort. Significant differences in CBC parameters were observed between age groups for neutrophils, lymphocytes, monocytes, and hemoglobin (Figures 4E–H, respectively). Parameters that did not significantly differ between the age groups were basophil, RBC, and platelet numbers, as well as MPV (data not shown).

#### Serum Chemistry

**Figure 5:**
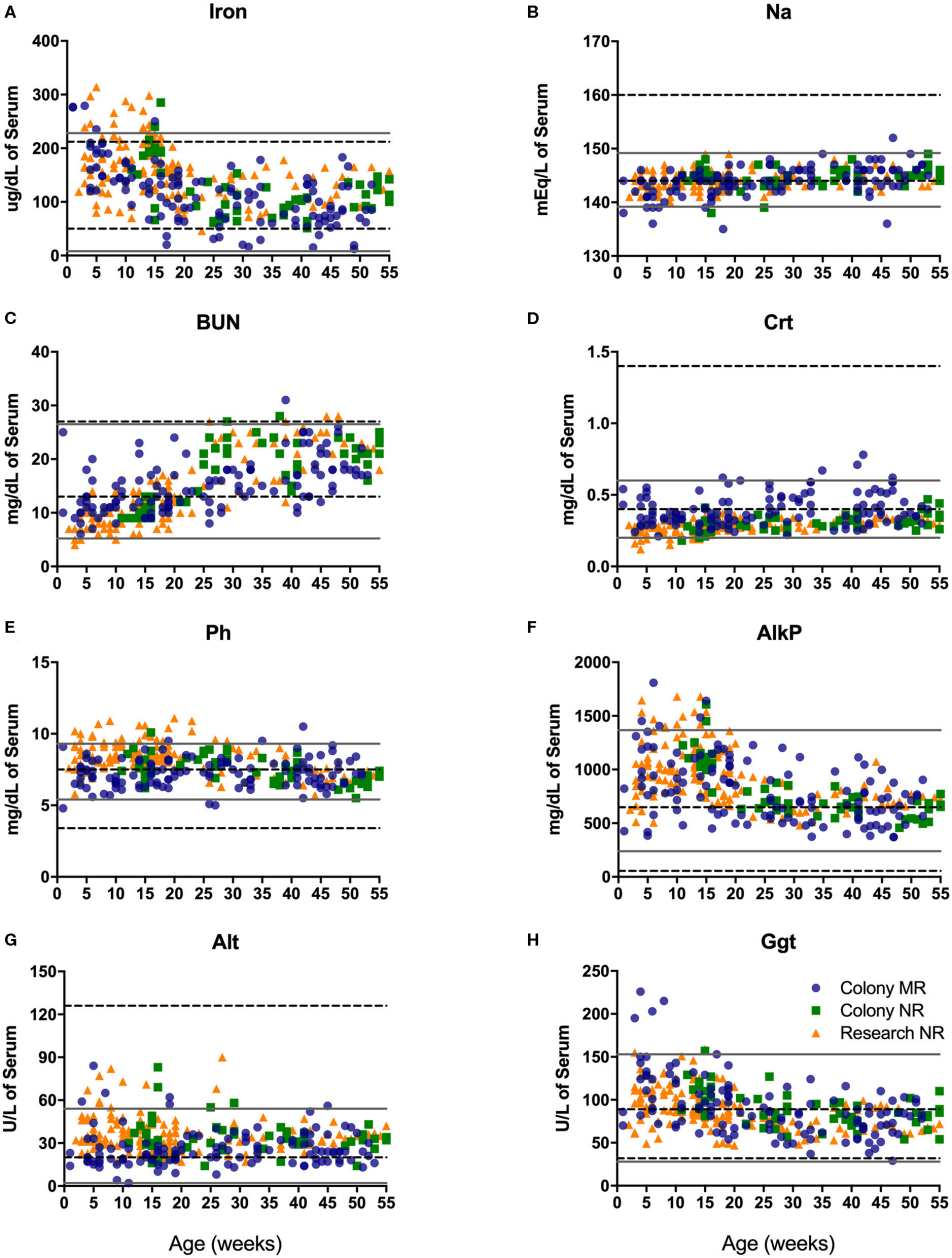
Clinical reference ranges (calculated as ±2 st. dev. values from the mean) for select serum chemistry parameters illustrate differences between pediatric <1yr old (gray solid lines) and adult (dotted black lines) rhesus macaques. Data points were plotted at age of sampling from Colony MR animals (blue circles), Colony NR animals (green squares), and Research NR animals (orange triangles). Parameters analyzed consisted of iron **(A)**, sodium or Na **(B)**, blood urea nitrogen or BUN **(C)**, creatinine or Crt **(D)**, phosphorus or Ph **(E)** alkaline phosphatase or AlkP **(F)**, alanine aminotransferase or Alt **(G)**, and gamma glutamyl transferase or Ggt **(H)**.

Blood chemistry analysis is used to evaluate the overall health and function of major organs and organ systems. Compared to adult rhesus macaque in-house reference ranges, infants presented with slightly higher values for iron early after birth (Figure 5A), phosphorous (Ph; Figure 5E), alkaline phosphatase early in infancy (Figure 5F), and gamma glutamyl transferase (GGT; Figure 5H), as well as glucose, AST and LDH (not shown). In contrast, pediatric animals presented with lower than in-house adult ranges in levels of alanine amino transferase or ALT (Figure 5G), sodium (Na; Figure 5B), and creatinine (CRT; Figure 5D), as well as chloride, albumin, globulin, calcium, and bilirubin (data not shown). Interestingly, levels of blood urea nitrogen (BUN; Figure 5C) were much lower compared to adult ranges in early life (0–25 wks of age) and increased to adult levels by 1 year of age.

**Figure 6:**
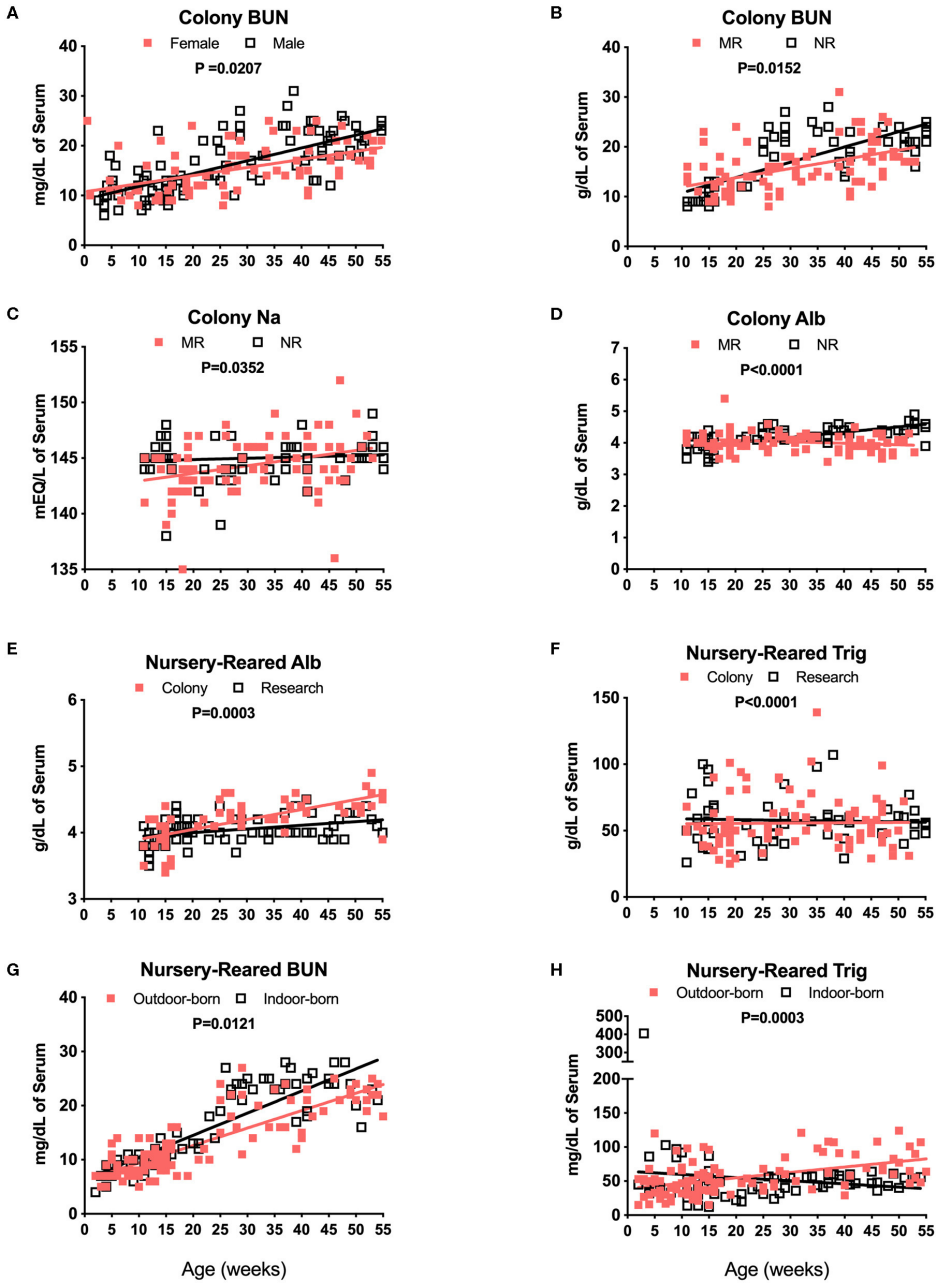
Analysis of blood chemistry values affected by breast milk (MR animals) vs. formula (NR animals), sex, and birth site. Linear regression and ANCOVA were was performed to compare slopes for male and female colony animal BUN data over age (Male: slope = 0.258, *R*^2^ = 0.4954; Female: slope = 0.1626, *R*^2^ = 0.2547) **(A)** and Colony MR and Colony NR animal BUN (MR: slope = 0.185, *R*^2^ = 0.2376; NR: slope = 0.3067, *R*^2^ = 0.5684) **(B)**. Comparisons over age were plotted to compare Colony NR and Colony MR animals for changes over age at sample collection time in levels of Na (MR: slope = 0.0687, *R*^2^ = 0.1139; NR: slope = 0.012, *R*^2^ = 0.009) **(C)** albumin (Alb, MR: slope = −0.0047, *R*^2^ = 0.0403; NR: slope = 0.0167, *R*^2^ = 0.4709) **(D)** and triglycerides (Trig, MR: slope = 0.0245, *R*^2^ = 0.0002; NR: slope = −0.0566, *R*^2^ = 0.0022) **(E)**. Colony NR and Research NR animals were compared over age for levels of Alb (Colony: slope = 0.015, *R*^2^ = 0.4552; Research: slope = 0.0056, *R*^2^ = 0.1387) **(F)**, and NR animals born indoors and outdoors were compared over age for BUN (Outdoor: slope = 0.3222, *R*^2^ = 0.7243; Indoor: slope = 0.4091, *R*^2^ = 0.7332) **(G)**, and Trig (Outdoor: slope = 0.7941, *R*^2^ = 0.2419; Indoor: slope = −0.4637, *R*^2^ = 0.0217) **(H)**.

Blood chemistry results that are known to be affected by diet were compared among the different pediatric groups. For example, breast milk from rhesus macaques and humans comprises a similar overall composition, with the exception of some oligosaccharides not present in rhesus macaque milk (25). Nursery-reared pediatric animals at the TNPRC receive Similac Advance Optigro formula (Abbott Laboratories) designed to match the essential nutrients of (human) breast milk, with additional ingredients used to optimize nutritional content for growing babies (i.e., Docosahexaenoic acid), a formulation that falls within the European Society for Pediatric Gastroenterology Hepatology and Nutrition (ESPGHAN) guidelines (26). For MR (breast-fed) infants, levels in BUN over time appeared to be influenced by sex with a significant difference in slopes of results between males and females (Figure 6A, *P* = 0.0207). There also were significant differences in slopes between Colony MR (breast-fed) and Colony NR (formula-fed) animals for levels of BUN (Figure 6B, *P* = 0.0152), Na (Figure 6C, *P* = 0.0352) and serum albumin (Figure 6D, *P* < 0.0001) over age through 55 weeks. Additional significant differences between MR and NR animals were observed (but data plots were not shown) for levels of glucose (*P* = 0.0031), phosphorus (*P* = 0.0103), globulin (*P* = 0.0033), calcium (*P* = 0.0407), and Alt (*P* = 0.0411) over time through 55 weeks of age. However, even between Colony NR vs. Research NR animals provided identical feeding regimens, there were significant differences across age groups for albumin (Figure 6E, *P* = 0.0003), triglycerides (Figure 6F, *P* < 0.0001) and glucose (data not shown, *P* = 0.0099). This suggests that both social environment and research intervention may impact blood chemistry levels during pediatric development that affect “normal” reference ranges. In addition, nursery-reared animals born outdoors have lower BUN (Figure 6G, *P* = 0.0121) and higher triglycerides (Figure 6H, *P* = 0.0003) with increasing age compared to those born indoors.

**Figure 7:**
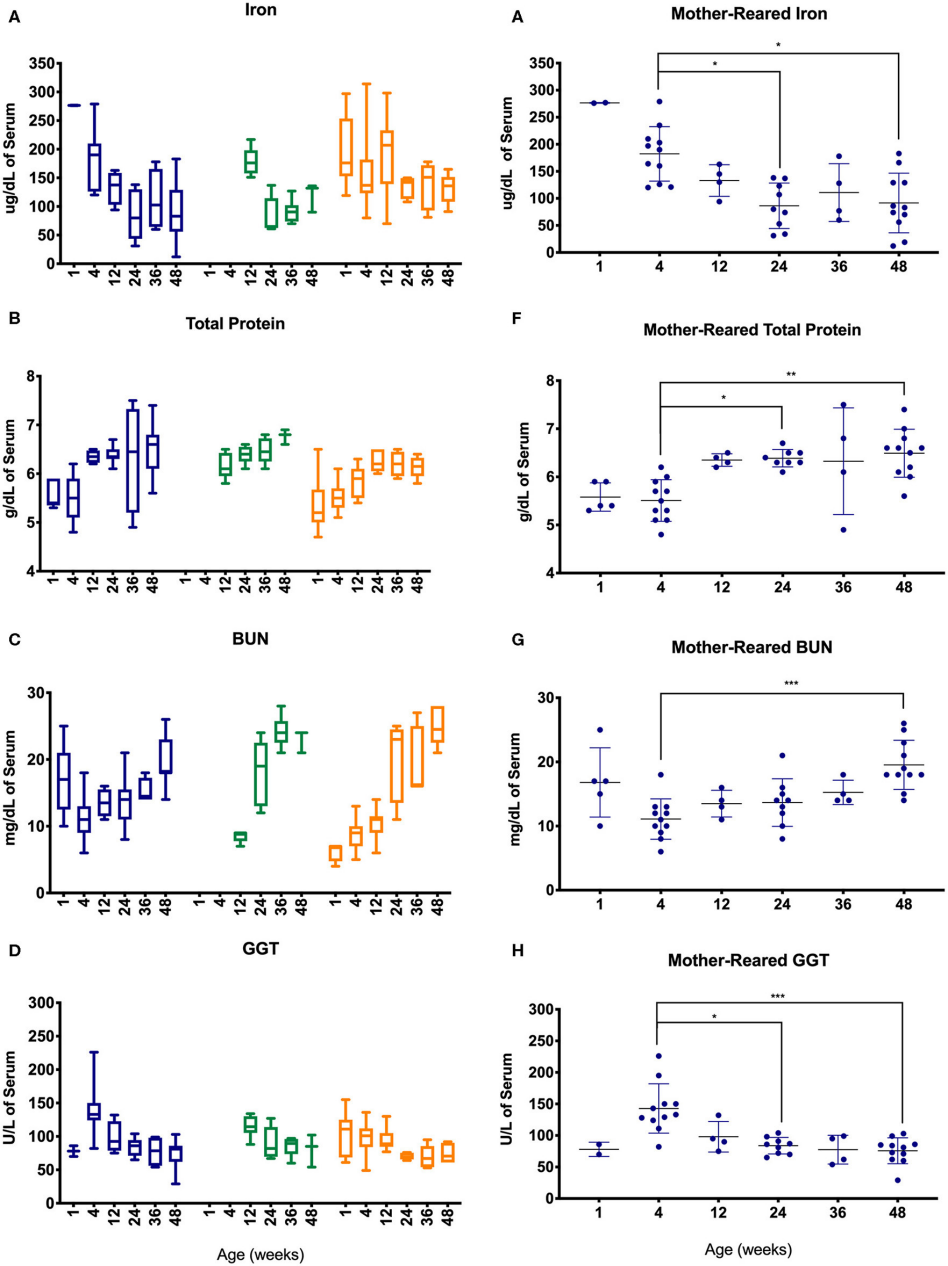
Trends in blood chemistry values in pediatric animals through the first year of life. Age binning was completed for samples from animals with average ages of 1 week (0–2 weeks old), 4 weeks (3–5 weeks old), 12 weeks (11–13 weeks old), 24 weeks (22–26 weeks old), 36 weeks (34–38 weeks old), and 48 weeks (46–50 weeks old). Data were shown for iron **(A)**, total protein **(B)**, BUN **(C)**, and GGT **(D)** from the Colony MR (blue), Colony NR (green), and Research NR (orange). **(A–D)** were plotted using box and whiskers depicting median, minimum and maximum values and **(E–H)** applied statistical analyses of results (mean ± st. dev.) from the Colony MR animals using Kruskal-Wallis test coupled with Dunn's multiple comparisons to illustrate how CBC parameters vary with age. *P < 0.05; **P < 0.01; ***P < 0.001.

Serum chemistry results from pediatric age-binned groups of animals were then plotted to demonstrate effects of age stratification (Figures 7A–D) and results from MR animals were further analyzed given that more sampling data were available from a larger number of animals over a greater age range (Figures 7E–H). Iron levels were higher in younger age groups among Colony MR, Colony NR, and Research NR pediatric animals (Figure 7A) and then declined significantly between 4 weeks of age to 24 and 48 weeks of age in the MR group (Figure 7E). A similar pattern was observed for GGT (Figures 7D,H) with higher values at 4 weeks of age followed by declining levels during the remaining first year of life (Figures 7D,H). In contrast, total protein (Figures 7B,F) and BUN (Figures 7D,G) levels increased after 4 weeks of age.

### Blood Cell Immunophenotyping

**Figure 8:**
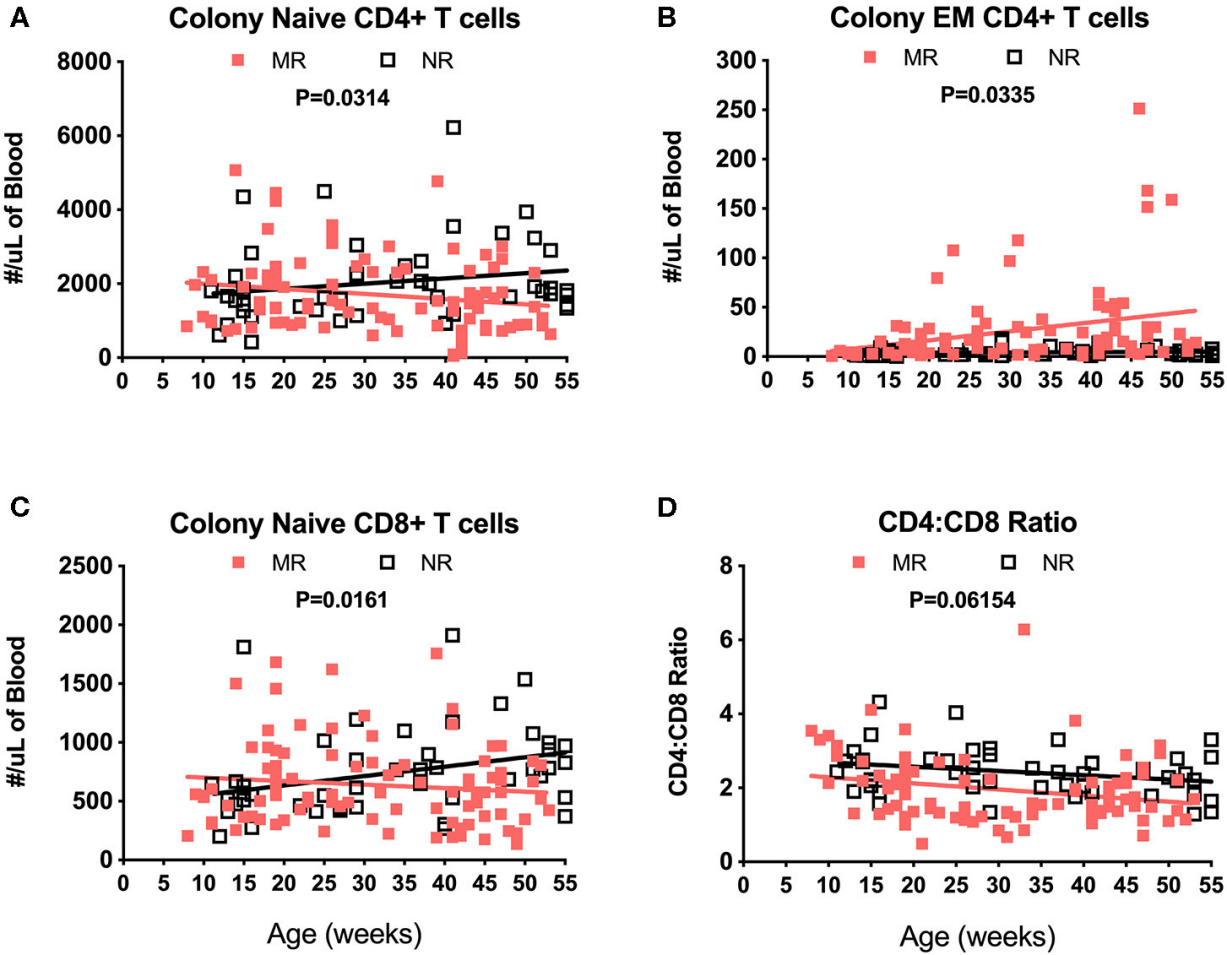
Linear regression and ANCOVA of select immunophenotyping parameters comparing results between Colony MR and Colony NR rhesus macaques during the first year of life for numbers of; naïve CD4+ T cells **(A)**, effector memory (EM) CD4+ T cells **(B)**, naïve CD8+ T cells **(C)**, and CD4:CD8 T cell ratios **(D)** Results were plotted by age at time of collection.

Immunophenotyping of peripheral blood mononuclear cells (PBMC) from the pediatric macaque cohorts is summarized in Table 2 and additional data stratified by rearing condition were shown in Tables S4A,B. The proposed ranges for the colony and research pediatric animals were based on means ± 2 Std. Dev. values of each cell type. Ranges in immune cell levels were quite broad, particularly in the case of the colony animals and suggested dynamic shifts occurring during the first year of life. Regression analyses applied to data from Colony MR and Colony NR animals revealed significant differences in slopes for numbers of naïve CD4+ T cells (Figure 8A, *P* = 0.0314), effector memory (EM) CD4+ T cells (Figure 8B, *P* = 0.0335), and naïve CD8+ T cells (Figure 8C, *P* = 0.0161). In contrast, the slopes for CD4:CD8 ratios over time did not differ significantly between these groups (Figure 8D, *P* = 0.06154). Significant differences also were observed in percentages of naïve CD4+ T cells (*P* = 0.0098), EM CD4+ T cells (*P* = 0.0293), central memory (CM) CD4+ T cells (*P* = 0.0165), and naïve CD8+ T cells (*P* = 0.0497) between Colony MR and Colony NR animals (data not shown). No significant differences between Colony MR and Colony NR animals were noted over the first year of life in the slopes of numbers and percentages of B cells, NK cells, monocyte subtypes and DC subtypes (not shown).

**Table 2:** Immunophenotyping of peripheral blood mononuclear cells in pediatric colony and research rhesus macaques.

**Parameter**	Proposed range (colony)^*^	Proposed range (research)^*^	**Units**
CD3+	0–7,857	438–5,490	#cells/uL blood
CD20+	0–5,339	0–2774.6	#cells/uL blood
NK	0–1477.5	0–613.6	#cells/uL blood
CD4+	0–4,807	112.6–3793.4	#cells/uL blood
Total CD14+	0–1403.5	61.1–682.7	#cells/uL blood
CD14–CD16+	0–364.5	0–147	#cells/uL blood
CD14+CD16–	0–1242.8	51.8–620.2	#cells/uL blood
CD1c+	0–82.5	0–38	#cells/uL blood
CD123+	0–29.6	0–14.2	#cells/uL blood
Naïve CD4+	0–3,849	0–3386.4	#cells/uL blood
Central memory CD4+	0–1181.5	96.4–472.4	#cells/uL blood
Effector memoryCD4+	0–83	0–37.6	#cells/uL blood
CD8+	0–2,769	0–1,932	#cells/uL blood
Naïve CD8+	0–1355.2	20.5–1091.7	#cells/uL blood
Central memory CD8+	0–457.3	1.3–179.9	#cells/uL blood
Effector memory CD8+	0–1,291	0–817.3	#cells/uL blood
%CD4+	43–80.5	44.4–88.5	% of CD3
%CD8+	13.8–47.6	9.5–48.1	% of CD3
CD4:CD8 Ratio	0.4–4.2	0–5.7	#CD4+/#CD8+

* *range values were determined from the mean +/– 2 standard deviations. A negative value for the lower range was designated as 0*.

**Figure 9:**
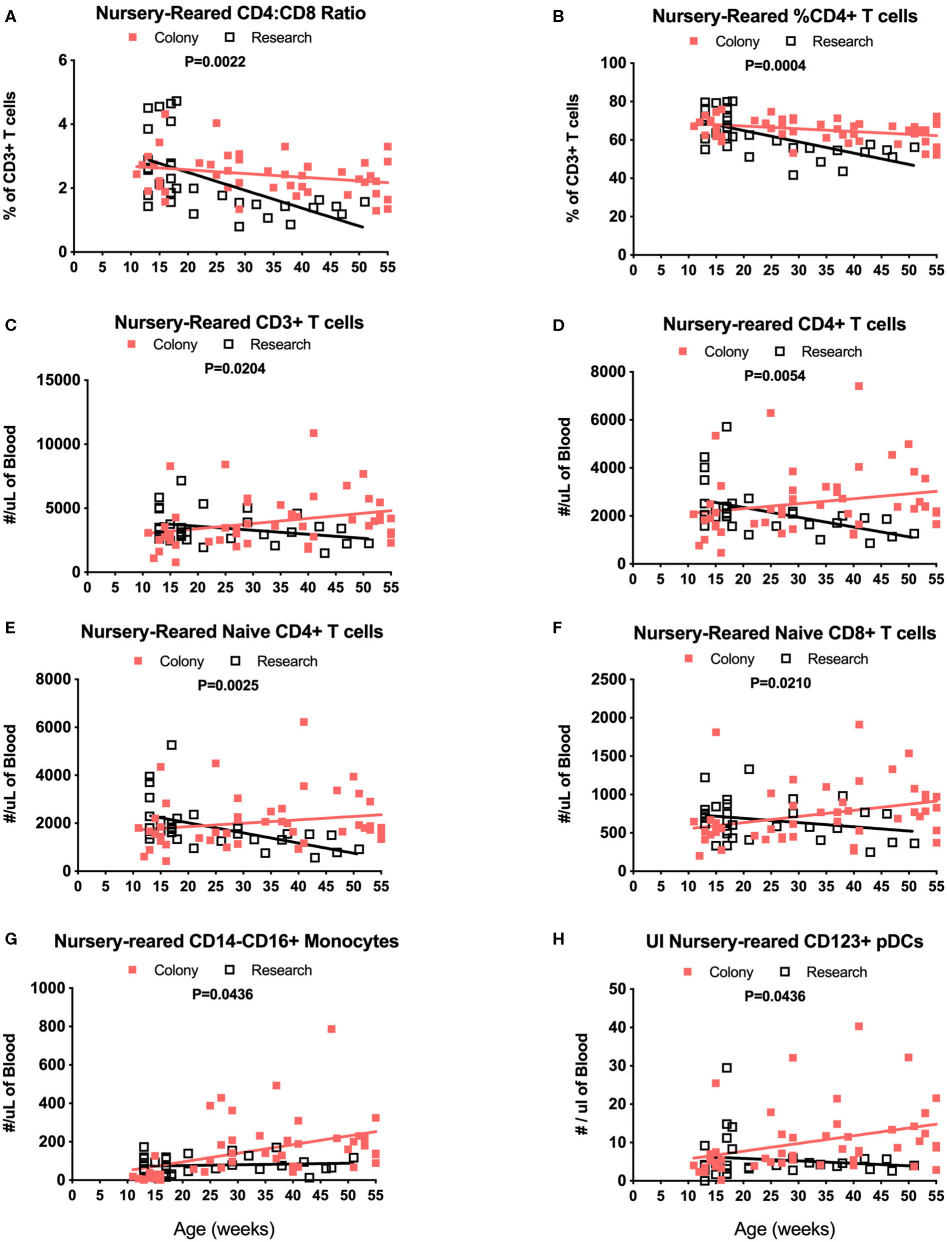
Linear regression and ANCOVA analyses of selected immunophenotyping parameters for comparison between Colony NR and Research NR animals over age during the first year of life for; CD4:CD8 ratios **(A)**, percent of CD4+ T cells **(B)**, number of CD3+ T cells **(C)**, number of CD4+ T cells **(D)**, number of naïve CD4+ T cells **(E)**, number of naïve CD8+ T cells **(F)**, number of non-classical monocytes **(G)**, and number of plasmacytoid dendritic cells (pDCs) **(H)**.

Among NR groups of animals, there were significant differences between colony and research animals in slopes of CD4:CD8 ratios over time (Figure 9A, *P* = 0.0022) as well as in percentages of CD4+ T cells (Figure 9B, *P* = 0.0004). There also were significant differences between the Colony and Research NR animals over the first year of life in slopes of percentages of CD8+ T cells (*P* = 0.0003), naïve CD4+ T cells (*P* = 0.0026), CM CD4+ T cells (*P* = 0.0086), EM CD4+ T cells (*P* < 0.0001), naive CD8+ T cells (*P* = 0.0002), and EM CD8+ T cells (*P* < 0.0001) (data not shown). Slopes in absolute numbers of cells over the first year of life were significantly different between these colony and research NR groups for CD3+ T cells (Figure 9C, *P* = 0.0204), CD4+ T cells (Figure 9D, *P* = 0.0054), naïve CD4+ T cells (Figure 9E, *P* = 0.0025), EM CD4+ T cells (*P* = 0.0024, data not shown), naïve CD8+ T cells (Figure 9F, *P* = 0.0210), non-classical monocytes (Figure 9G, *P* = 0.0436), and CD123+ plasmacytoid DCs (pDCs) (Figure 9H, *P* = 0.0436).

**Figure 10:**
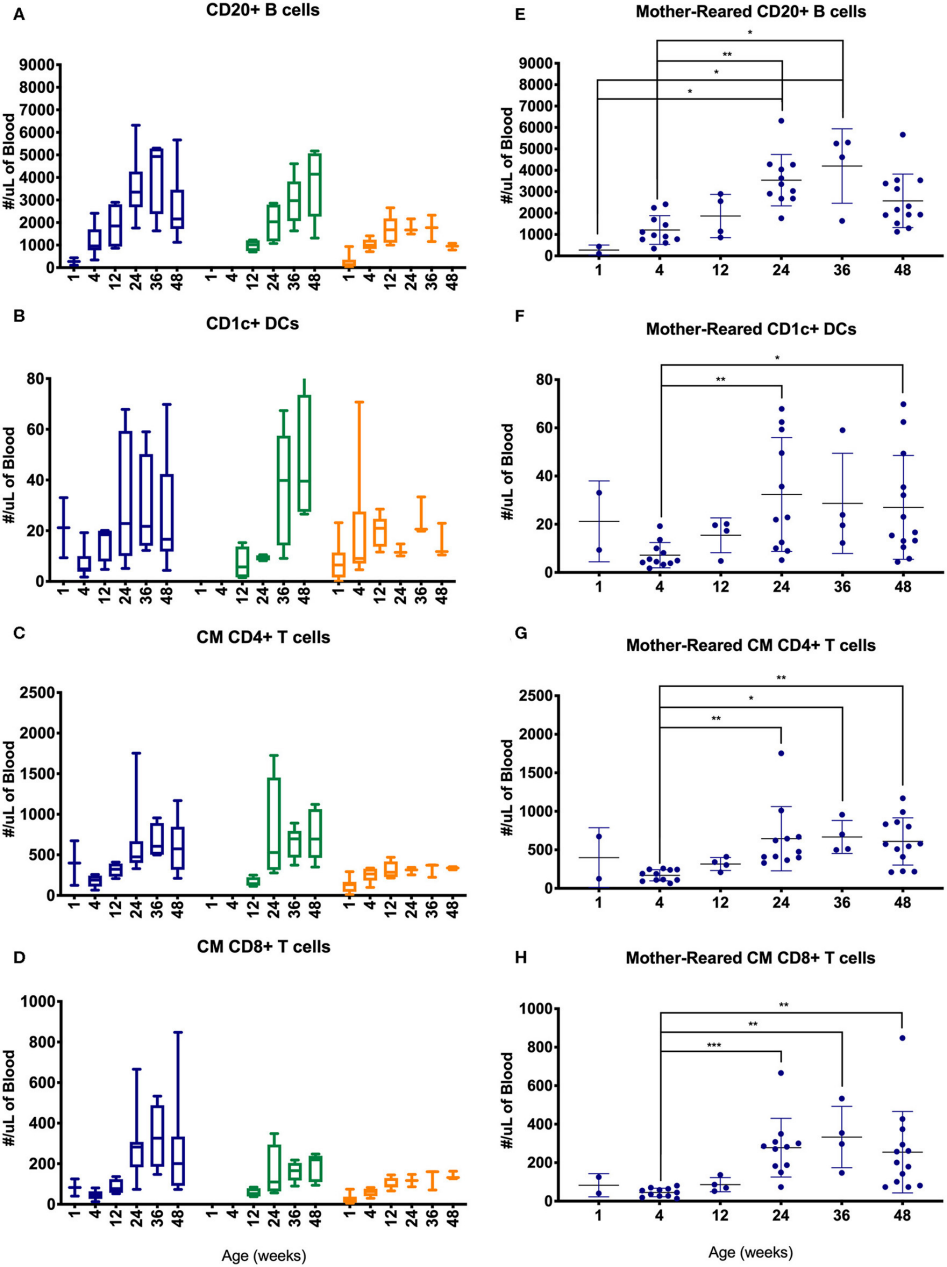
Comparison of selected PBMC immunophenotyping parameters. Age binning was completed for samples from animals with average ages of 1 week (0–2 weeks old), 4 weeks (3–5 weeks old), 12 weeks (11–13 weeks old), 24 weeks (22–26 weeks old), 36 weeks (34–38 weeks old), and 48 weeks (46–50 weeks old) of the Colony MR cohort (blue), Colony NR (green), and Research NR cohort (orange). Results are shown for; **(A,E)** CD20+ B cells, **(B,F)** CD1c+ dendritic cells, **(C,G)** CM CD4+ T cells, and **(D,H)** CM CD8+ T cells. Panels **(A–D)** were plotted using box and whiskers depicting median, minimum and maximum values and panels **(E–H)** applied statistical analyses of results (mean ± st. dev.) from the Colony MR animals using Kruskal-Wallis test coupled with Dunn's multiple comparisons to illustrate how CBC parameters vary with age. *P < 0.05; **P < 0.01; ***P < 0.001.

Lymphocyte counts generally increased during the first year of life with some differences between cohorts of MR and NR rhesus macaques (Figure 10). Specifically, B cells, CD1c+ DCs, CM CD4+ T cells and CM CD8+ T cells increased over the first 6 months, especially in the colony groups compared to research animals (Figures 10A–D). In the Colony MR group (from which a larger number of animal specimens were evaluated) there were significantly higher numbers of these cell populations at 24 weeks of age compared to 4 weeks of age, with cell counts stabilizing between 24 and 48 weeks of life.

### Cytokine Data

Evaluation of circulating cytokine levels offers supportive information to blood chemistry, CBC, and PBMC immunophenotyping data for monitoring immune development during the first year of life. Data were pooled from the three rearing groups and stratified by age groups (Table 3). Data from adults aged 5–15 years were from our previously published results (27) and supplemented with results from additional adult colony animals that were evaluated since then. Analyses of adult macaques showed that levels in pro-inflammatory cytokines IFNγ, IL-1β, IL-6, IL12/23p40, IL-15, MIF, sCD40L, and TNFα significantly correlated with age. Similarly, chemokine levels of IL-8, MCP-1, MIP-1α, and MIP-1β significantly correlated with age. Of the anti-inflammatory cytokines and growth factors tested, levels of IL1ra, IL-4 and IL5, EGF, G-CSF, IL2, and TGFα also showed significant correlations with age (27).

**Table 3:** Pediatric Plasma Cytokines.

				**95% CI**				
		**Mean**	***SD***	**Lower**	**Upper**	**Median**	**Range**	**Number of samples**
**GM-CSF (pg/mL)**								
	<1 wk	9.903	7.085	5.143	14.66	6.89	2.23–23.1	11
	1–2 wks	16.86	6.274	12.65	21.08	15.08	7.59–25.89	11
	12–16 wks	12.95	7.805	9.393	16.5	10.84	3.67–31.89	21
	20–24 wks	8.302	4.923	5.326	11.28	9.31	0.59–15.7	13
	52 wks	14.16	6.935	8.364	19.96	12.01	5.7–27.72	8
	5–15yrs	66.09	213.4	5.452	126.7	4.56	0–1,086	50
**TGFα** **(pg/mL)**								
	<1 wk	5.905	3.663	3.443	8.366	4.62	1.82–13.74	11
	1–2 wks	6.107	2.116	4.686	7.529	5.81	2.78–9.68	11
	12–16 wks	11.59	9.646	6.631	16.55	5.74	2.5–31.89	17
	20–24 wks	5.097	3.518	3.149	7.046	4.58	1.55–14.95	15
	52 wks	8.01	3.588	5.01	11.01	8.965	2.22–12.59	8
	5–15yrs	7.635	6.788	5.404	9.866	5.94	0–28.68	38
**G-CSF (pg/mL)**								
	<1 wk	45.68	35.47	21.86	69.51	35.22	10.65–114.6	11
	1–2 wks	66.81	35.54	42.93	90.68	59.47	22.14–135.5	11
	12–16 wks	36.59	17.91	27.68	45.5	33.64	16.73–79.76	18
	20–24 wks	33.05	26.01	19.68	46.42	31.41	0–100.4	17
	52 wks	38.69	26.37	16.65	60.73	38.34	11.43–76.1	8
	5–15yrs	77.33	87.95	52.07	102.6	50.54	0–272.8	49
**IFNγ** **(pg/mL)**								
	<1 wk	9.208	9.413	2.884	15.53	7.64	0–23.14	11
	1–2 wks	19.59	11.92	11.59	27.6	19.17	0–37.83	11
	12–16 wks	24.45	18.94	15.03	33.87	17.46	0–61.6	18
	20–24 wks	16.04	14.11	8.782	23.29	18.72	0–40.82	17
	52 wks	21.23	12.17	11.05	31.4	18.55	8.36–47.69	8
	5–15yrs	27.35	23.64	20.64	34.07	22.6	0–78.09	50
**IL-2 (pg/mL)**								
	<1 wk	41.92	11.78	34.01	49.84	41.21	22.49–61.77	11
	1–2 wks	43.82	15.78	33.22	54.43	39.53	20.16–75.84	11
	12–16 wks	28.91	8.37	24.45	33.37	27.71	13.88–44.39	16
	20–24 wks	28.14	11.69	22.13	34.15	25.63	13.43–55.52	17
	52 wks	23.4	6.217	18.2	28.59	21.32	17.1–37.05	8
	5–15yrs	72.5	71.21	52.04	92.95	44	1.78–234.1	49
**IL-10 (pg/mL)**								
	<1 wk	85.75	30.7	65.13	106.4	91.34	35.06–128.9	11
	1–2 wks	123	57.6	84.26	161.6	103.9	56.84–252.6	11
	12–16 wks	82.07	31.25	66.53	97.61	77.73	40.86–160.1	18
	20–24 wks	64.9	37.21	45.77	84.03	61.91	22.73–183	17
	52 wks	74.02	29.73	49.17	98.88	65.84	31.55–126.2	8
	5–15yrs	4.556	5.425	3.03	6.081	2.64	0–25.93	51
**IL-15 (pg/mL)**								
	<1 wk	15.31	6.519	10.93	19.69	12.7	9.31–30.28	11
	1–2 wks	12.01	3.051	9.965	14.06	12.15	6.33–17.47	11
	12–16 wks	10.83	2.222	9.642	12.01	10.32	7.33–14.86	16
	20–24 wks	7.191	2.477	5.761	8.621	6.76	1.82–11.04	14
	52 wks	9.888	2.854	7.501	12.27	10.32	5.57–14.04	8
	5–15yrs	11.56	9.229	8.939	14.18	8.115	1.24–40.49	50
**sCD40L (pg/mL)**								
	<1 wk	2,354	1,982	935.7	3,771	1,968	255.5–6,280	10
	1–2 wks	1,014	631	528.7	1,499	951.9	226.6–1,876	9
	12–16 wks	831.4	492.7	558.6	1,104	750.6	247.9–1,773	15
	20–24 wks	713.6	513.1	449.8	977.4	549.3	60.53–1,827	17
	52 wks	335	284.5	71.85	598.1	296.5	61.47–784.1	7
	5–15yrs	1,965	3,225	919.7	3,011	548.1	66.83–10,000	39
**IL-17 (pg/mL)**								
	<1 wk	1.735	0.8546	1.161	2.31	1.63	0.76–3.51	11
	1–2 wks	2.966	1.604	1.732	4.199	2.38	1.08–5.19	9
	12–16 wks	1.573	0.685	1.113	2.033	1.46	0.8–3.31	11
	20–24 wks	1.628	0.8263	1.103	2.153	1.32	0.64–3.36	12
	52 wks	1.179	0.3899	0.8528	1.505	1.13	0.54–1.69	8
	5–15yrs	3.079	6.146	1.333	4.826	1.04	0–30.32	50
**IL-1ra (pg/mL)**								
	<1 wk	45.7	19.9	30.4	60.99	37.61	28.16–82.87	9
	1–2 wks	35.85	13.3	26.34	45.37	36.98	16.47–55.85	10
	12–16 wks	30.18	10.63	24.29	36.07	26.95	15.08–53.95	15
	20–24 wks	31.58	16.71	22.33	40.83	33.65	9.77–62.31	15
	52 wks	23.61	6.197	17.88	29.34	20.34	18.24–34.16	7
	5–15yrs	218.3	385.6	108.7	327.9	35.06	0–1,774	50
**IL-13 (pg/mL)**								
	<1 wk	33.36	9.062	27.27	39.45	35.32	17.55–46.58	11
	1–2 wks	44	11.6	36.2	51.79	43.23	28.23–65.5	11
	12–16 wks	46.68	17.9	36.77	56.6	46.33	27.84–89.72	15
	20–24 wks	45.17	22.19	32.89	57.46	37.87	13.93–91.1	15
	52 wks	39.74	6.744	34.1	45.38	40.98	31.33–48.42	8
	5–15yrs	2.905	3.895	1.642	4.168	2.5	0–23.78	39
**IL-4 (pg/mL)**								
	<1 wk	20.01	24.83	3.335	36.69	16.22	0–75.79	11
	1–2 wks	58.3	81.14	3.793	112.8	8.99	0–214.9	11
	12–16 wks	8.501	8.973	3.32	13.68	7.895	0–26.43	14
	20–24 wks	21.63	21.3	8.759	34.51	16.22	0–65.36	13
	52 wks	30.15	36.77	−0.5928	60.89	22.6	0–106.2	8
	5–15yrs	84.95	153.7	41.28	128.6	0	0–396.5	50
**IL-1β** **(pg/mL)**								
	<1 wk	1.836	0.9981	1.166	2.507	1.94	0.35–3.41	11
	1–2 wks	3.33	1.338	2.431	4.229	3.1	1.77–6.02	11
	12–16 wks	2.733	1.06	2.168	3.297	2.75	1.34–4.91	16
	20–24 wks	1.844	1.01	1.26	2.427	1.67	0.81–4.22	14
	52 wks	2.443	0.8683	1.717	3.168	2.37	1.02–3.95	8
	5–15yrs	10.01	14.25	5.965	14.06	3.205	0–44.84	50
**IL-5 (pg/mL)**								
	<1 wk	6.608	4.863	3.129	10.09	5.19	1.8–18	10
	1–2 wks	10.07	4.835	6.825	13.32	8.89	4.13–18.23	11
	12–16 wks	7.263	2.561	5.946	8.58	8.07	3.33–10.76	17
	20–24 wks	7.178	4.843	4.496	9.86	6.28	1.55–19.88	15
	52 wks	5.361	1.895	3.609	7.114	5.42	1.86–7.46	7
	5–15yrs	2.932	4.465	2.012	3.851	1.2	0–19.77	93
**IL-6 (pg/mL)**								
	<1 wk	4.376	2.751	2.528	6.224	4.48	0.83–9.15	11
	1–2 wks	4.443	2.098	3.034	5.852	4.33	1.73–8.45	11
	12–16 wks	2.828	1.553	2.001	3.655	2.475	0.93–6.85	16
	20–24 wks	2.198	1.717	1.161	3.236	2.07	0.22–6.51	13
	52 wks	2.576	1.06	1.69	3.463	2.405	0.97–3.95	8
	5–15yrs	3.591	4.166	2.382	4.801	2.32	0–16.15	48
**IL-8 (pg/mL)**								
	<1 wk	233.1	106.7	143.9	322.3	243.8	52.3–374.2	8
	1–2 wks	734.5	730.2	243.9	1,225	478.6	37.28–2,430	11
	12–16 wks	329.8	207.1	223.3	436.3	283.7	77.06–747.5	17
	20–24 wks	184.3	125.5	111.8	256.7	147.4	36.68–416.9	14
	52 wks	192.4	96.2	111.9	272.8	189.7	94.38–343.4	8
	5–15yrs	1,084	1,625	622.2	1,546	452.8	0–6,827	50
**TNFα** **(pg/mL)**								
	<1 wk	48.95	33.77	26.26	71.64	39.64	12.69–113.1	11
	1–2 wks	91.2	31.58	69.98	112.4	82.58	47.41–137.4	11
	12–16 wks	88.2	33.86	70.79	105.6	92.95	38.75–149.9	17
	20–24 wks	69.45	47.06	43.39	95.51	58.49	12.11–168.8	15
	52 wks	82.66	37.46	51.34	114	79.56	37.01–150.5	8
	5–15yrs	26.54	33.83	16.92	36.15	13.41	0–107.2	50
**IL-12/23p40 (pg/mL)**								
	<1 wk	27.77	24.62	11.23	44.32	20.51	0–71.75	11
	1–2 wks	60.06	27.07	41.87	78.24	53.12	21.09–98.56	11
	12–16 wks	41.53	15.68	32.06	51.01	39.67	19.72–67.79	13
	20–24 wks	49.59	50.48	19.08	80.1	34.92	0–149	13
	52 wks	43.98	23.64	24.21	63.74	40.05	16.98–96.27	8
	5–15yrs	239.1	318.9	148.4	329.7	90.52	12.63–1,148	50
**MIP-1α** **(pg/mL)**								
	<1 wk	19.58	6.885	14.95	24.2	22.25	8.29–27.5	11
	1–2 wks	29.2	8.947	23.19	35.21	27.8	18.17–47.92	11
	12–16 wks	30.78	14.42	22.79	38.76	25.99	15.61–66.18	15
	20–24 wks	22.09	6.513	17.95	26.22	23.2	10.21–34.98	12
	52 wks	23.83	6.502	18.39	29.26	23.63	16.54–35.77	8
	5–15yrs	26.61	21.3	20.55	32.66	25.93	0–65.25	50
**MIP-1β** **(pg/mL)**								
	<1 wk	7.9	5.136	4.449	11.35	7.3	0.91–16.02	11
	1–2 wks	13.71	5.369	10.1	17.32	13.48	6.99–22.01	11
	12–16 wks	10.75	5.374	8.077	13.42	10.4	0–21.26	18
	20–24 wks	6.772	2.258	5.408	8.137	6.32	3.14–10.05	13
	52 wks	12.07	10.47	2.387	21.76	8.23	1.72–34.25	7
	5–15yrs	18.33	17.84	13.26	23.4	13.11	0–75.98	50
**MCP-1 (pg/mL)**								
	<1 wk	707.2	371.7	457.5	957	529.7	382–1,508	11
	1–2 wks	610.3	375.7	357.8	862.7	515.5	228.6–1,602	11
	12–16 wks	284	75.88	245	323	265.6	150.3–429.3	17
	20–24 wks	229	102.1	170	288	196.3	93.83–450.3	14
	52 wks	252.3	81.48	184.2	320.4	230.7	160.7–422.5	8
	5–15yrs	314.2	222.9	250.9	377.6	250.3	88.16–1,345	50
**VEGF (pg/mL)**								
	<1 wk	62.05	35.04	38.51	85.59	46.18	30.55–132.8	11
	1–2 wks	88.83	70.74	41.31	136.4	59.36	20.5–206.3	11
	12–16 wks	52.15	35.77	29.42	74.87	41.44	6.24–115.2	12
	20–24 wks	44.56	43.2	17.11	72.01	33.78	0–152.7	12
	52 wks	33.81	12.87	23.05	44.57	31.34	19.4–58.61	8
	5–15yrs	55.61	132.5	17.96	93.27	0	0–707.9	50
**IL-18 (pg/mL)**								
	<1 wk	83.52	38.12	56.25	110.8	93.16	0–122.6	10
	1–2 wks	121.1	61.34	79.91	162.3	103.2	51.19–249.6	11
	12–16 wks	64.18	33.75	46.82	81.53	51.19	22.84–130.3	17
	20–24 wks	72.78	72.22	36.86	108.7	62.78	0–276.3	18
	52 wks	34.27	31.94	7.57	60.97	30.69	0–90.88	8
	5–15yrs	NA						0

Among the pediatric groups of rhesus macaques, levels of several cytokines in comparison to those detected in the adults (Table 3). In particular, pediatric animals during the first year of life expressed strikingly higher levels of IL-10, IL-13, and TNFα and conversely, expressed dramatically lower levels of IL-1β, IL-8, and IL12/23p40 compared to adults. The lower pro-inflammatory cytokines (IL-1β and IL12/23p40) and higher anti-inflammatory cytokines (IL-10 and IL-13) in the pediatric animals supports a skewing toward Th2 immunity, lower inflammation, and tolerance when compared to adults.

**Figure 11:**
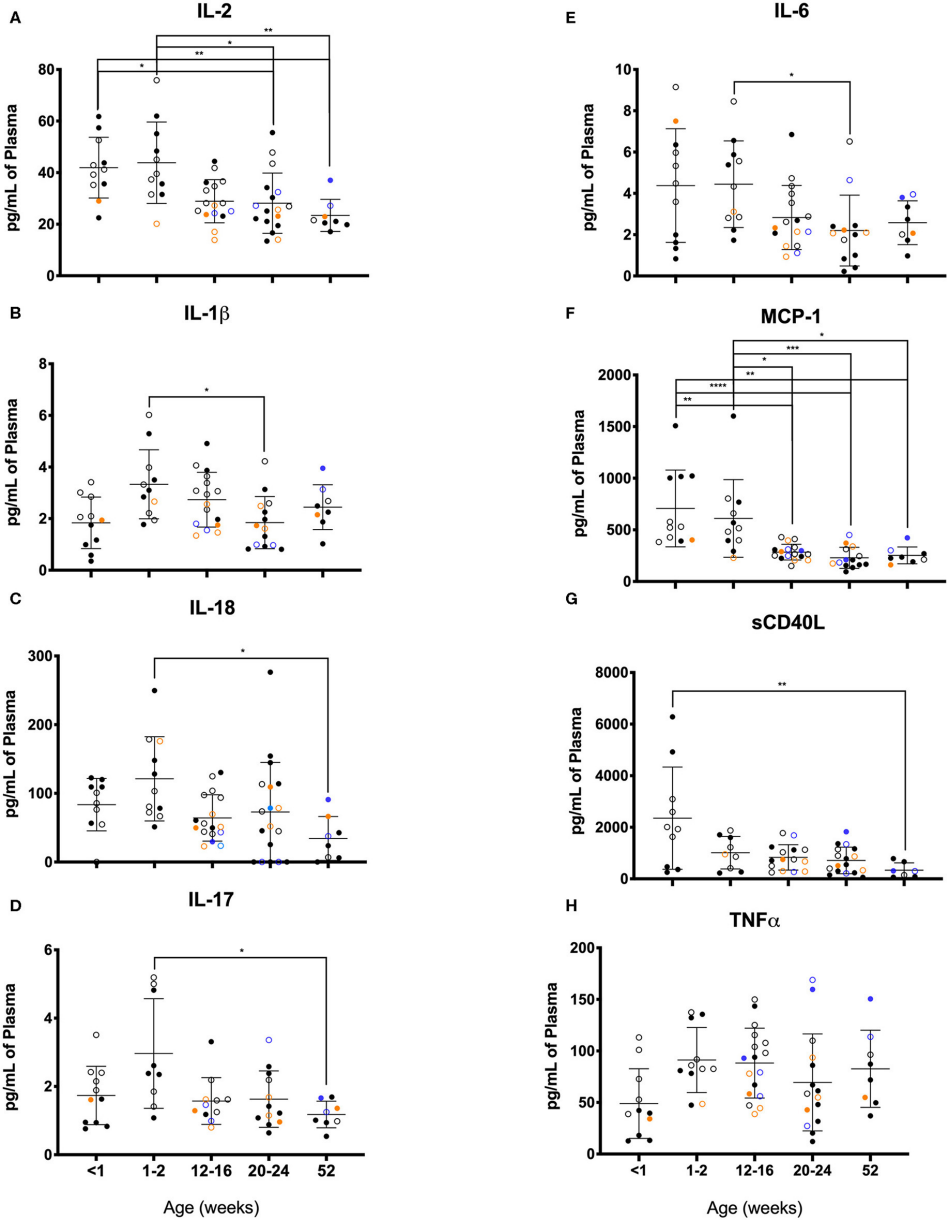
Plasma cytokine concentrations were compared among age groups of pediatric rhesus macaques during the first year of life. Plotted data are shown for **(A)** IL-2, **(B)** IL-1β, **(C)** TNFα, **(D)** IL-17, **(E)** IL-6, **(F)** MCP-1, **(G)** sCD40L, and **(H)** IL-18. Results represent Research NR (black circles), Colony NR (blue circles), and Colony MR (orange circles) animals as well as those born outdoors (open circles) and indoors (filled circles). Results were plotted for each data point (mean ± st. dev. per age group). Statistical analyses were by based on results of age-binned samples in the Colony MR animals using the Kruskal-Wallis test coupled with Dunn's multiple comparisons test. *P < 0.05; **P < 0.01; ***P < 0.001; ****P < 0.0001.

As illustrated in Figure 11, clear age-related differences in cytokine results as well as from various rearing conditions or birth locations can be visualized. Due to small sample sizes in some groups, statistical comparisons were sometimes only between age groups and not between rearing or birthing conditions. Statistical comparisons between age groups within the first year of life demonstrated significant differences between the two youngest age cohorts that exhibited higher cytokines levels that then were lower in the remaining age groups of 12–16, 20–24, and 52 weeks. Specifically, in groups <2wks old compared to groups aged 20–52 weeks, there were statistically higher levels of IL-2 (Figure 11A), IL-1b (Figure 11B), IL-18 (Figure 11C), IL-17 (Figure 11D), IL-6 (Figure 11E), MCP-1 (Figure 11F), and sCD40L (Figure 11G), as well as IL-8, IL-15, IL-1ra, MIP-1β, GM-CSF, G-CSF, and IL-10 (data not shown). The remaining cytokines tested remained level during infancy at the ages tested and included TNFα (Figure 11H), IL-13, IL-4, MIP-1α, VEGF, TGFα, IFNγ, IL-5, and IL12/23p40 (data not shown). In addition, NR animals born outdoors appeared to express higher levels of IL-1β (Figure 11), IL-17 (Figure 11D), and TNFα (Figure 11H), but lower levels of MCP-1 (Figure 11F) compared to those born indoors. This suggests that environmental exposures affect early immunological development cytokine parameters.

## Discussion

The rhesus macaque pediatric model is valuable for studies that translate to human children (28, 29). In particular, rhesus macaque immune ontogeny throughout fetal development and soon after birth is extraordinarily similar to that seen in humans (30). Further, the impacts of stress, maternal health, diet, type of birth delivery, and environmental exposures on infant rhesus macaques and their greater disease susceptibility is comparable to that reported in human infants (31–34). Currently, however, there are no established clinical hematological reference ranges for assessing “normal” development in pediatric rhesus macaques. In some studies, selected clinical parameters such as percentages or ratios of cell subtypes have been reported but these depend on methodologies affecting denominator values that often vary between study sites. Absolute numbers of cells can offset these variations and therefore, relating ranges of absolute values over early age was one purpose of this study. In addition, this study sought to examine the CBC, blood chemistry, and circulating immune cell populations at different ages during the first year of life due to the rapid shifts that occur during development. This is in part to help mediate and clarify the designations applied to grouping young animals as neonates, infants, or juveniles that may be inconsistently applied in different studies. Many rhesus macaque studies use pre-infection data for comparison to experimental results at later stages without accounting for “normal” developmental shifts, and this requires careful inclusion of controls that are closely matched by age group. Furthermore, housing status, environmental exposure, sex, and nutrition source (formula vs. breast milk), among other factors, likely impact the rate of changes in clinical parameters. Thus, this study was intended to help standardize hematological, clinical chemistry, and immune cell parameters during the first year of life in rhesus macaques in support of veterinary clinical management as well as to improve interpretation of experimental research results for translation to human pediatric patients. Limitations of the study worth noting include that the number of individual samples are highest for the MR colony cohort, however the majority of Colony MR animals were sampled at a single occasion, while NR groups were sampled longitudinally, with most frequent access within the Research NR animals. Differences in group N, sampling frequency and effects of anesthetic events, may bias the data in statistical comparisons performed between groups. Additionally, the animals represented in this study were sampled from a single primate center. Ideally, pediatric animal cohorts from other primate centers would be included in order to account for differences that could occur between centers in environment, husbandry, diet, microbiome, etc., that would influence immunological development.

Important developmental events continue soon after birth in the blood and bone marrow based on shifting hematopoietic parameters (23), thereby reiterating the need for age-specific reference range values. Human newborns reportedly express higher WBC, RBC, platelets, and innate immune cells such as neutrophils and monocytes when compared to adults (35). Gestation time also may affect CBC values with pre-term human neonates exhibiting lower hematocrit, hemoglobin (HGB) and platelet count, along with higher MCV and mean corpuscular hemoglobin (MCH) compared to full-term human neonates (36). Cord blood as an alternate specimen to neonatal blood for evaluation has proven inconsistent between full-term and pre-term neonates, particularly regarding neutrophil counts (36, 37).

CBC values in pediatric rhesus macaques, as also reported for pediatric humans, were higher for innate cells and RBC (Figures 2A,B,F) compared to adults of both species (36, 37). Further differences in CBC values were noted between breast-fed and formula-fed rhesus infants suggesting early diet or source of nutrition, and perhaps housing environment, influenced neutrophil and lymphocyte levels (38). A study by Capitanio (39) stratified data from 595 rhesus infants by sex and rearing condition at ~15 weeks of age. The results reported higher lymphocytes and lower neutrophils in the indoor-housed groups compared to outdoor-housed groups as well as higher variability in numbers of monocytes and eosinophils among males housed in corn cribs. Here, we observed similar trends of higher lymphocytes but not lower neutrophil levels in MR compared to NR animal groups (Table 1). Mean CBC values of macaques aged ~12 weeks in our study (Figures 4A–D) were within the published 95% CI range of the ~15 week-old rhesus macaques in the Capitanio study (39) with exception of WBC, HGB, MCH, platelets and neutrophils that were lower in our animals. These divergences may reflect differences in sample size, ancestry, founder effects, SPF status, specimen collection practices, or that there occurs a critical hematologic development change between 12 and 15 weeks of age. A report by Fernie et al. (40) analyzed data from nine rhesus infants between the ages of 12 and 122 weeks of age after use of anesthetic or manual restraint for blood collection. The results demonstrated slight but consistently lower values for RBC, HGB, HCT, MCV, lymphocyte and monocyte levels after use of ketamine for sample collection. The reported CBC ranges of values in the Fernie study fell within our reported ranges, with the exception of neutrophil counts (0.8–12.87 × 10^3^/ul) that were higher than results from our animals (0.21–10.2 × 10^3^/ul), as well as MPV range of 9.8–14.4 fL in our animals compared to 8.1–11.4 fL reported in the previously published study (40). These discrepancies could reflect lower age groups, higher number of animals, or differences in rearing conditions of our study.

Parameters measured in serum chemistry analyses included electrolytes, proteins, lipids, and glucose levels, which can be used to detect perturbations such as dehydration, malabsorption, kidney disease, diabetes, liver damage, cardiomyopathies, inflammation or infection. The serum chemistry results and ranges of values in the rhesus macaques of this study were largely similar to those reported by Fernie et al. (40). Exceptions noted included the higher range of values for AST and ALT, and lower range values in our animals for BUN, creatinine, total bilirubin, and triglycerides in our results. These differences could be due to our much larger sample size, inclusion of samples from animals <12 weeks of age, or a discrepancy in animal diet or environment, as these could not be compared between studies.

Due to growth and biological development, particularly those that reflect bone development, muscle growth and metabolism, striking differences in serum chemistry parameters have been described between juveniles, adults and the elderly in humans and other species (41–43). For example, growth spurts in human children and adolescents (ages 3–17 years) correlated with increased Ca, phosphate, and AlkP levels that were likely related to bone formation. In addition, higher levels of Alb and total protein in children appeared to reflect growth and maturation of the liver, and higher Crt and urea levels likely were associated with increasing muscle mass. As a result of hormone synthesis during development, LDL and Trig parameters also were seen to increase with age (43). According to a study reported by Gomez et al. (44) age-related differences within human pediatric cohorts also were apparent for most serum biochemistry parameters, noting significant variances in newborns (<1-month-old) compared to older ages for Crt, iron, bilirubin, creatine kinase, and Ca. For only two parameters did they find any significant influence of sex on pediatric serum chemistry analysis, which included Trig and lactate dehydrogenase (LDH) (44). We also observed significant differences between MR rhesus macaques age groups <4 weeks compared to older ages for total protein, albumin, globulin, calcium, and bilirubin, serum iron, GGT, and BUN, however the only significant difference between sexes detected in serum chemistry was BUN.

Higher BUN during neonatal development may be attributed to increasing muscle mass and maturation of the kidneys during normal growth. Interestingly, BUN was the only serum chemistry parameter that was significantly different between sexes being higher in males, and may reflect faster maturation of the kidneys and in muscle mass than occurs for other sex-specific characteristics. Nursery-reared animals born outdoors however, exhibited lower BUN (Figure 6G, *P* = 0.0121) and higher triglycerides (Figure 6H, *P* = 0.0003) with increasing age through the first year of life compared to those born indoors. These differences may be due to early environmental effects or confounded by variables such as gestational age or maternal health, which were not controlled for in this study.

In addition to BUN, total serum protein increased with age in all groups during the first year of life and differed between MR and NR groups as noted by comparisons of slopes with a steeper incline in NR animals as well as in both colony and research cohorts. These differences could be attributed to formula composition. However, maternal presence (or lack thereof) and different energy expenditures between primarily indoor-housed vs. outdoor-housed animals cannot be ruled out and represent areas for further investigation. Additional significant differences found between MR animals ages <4 weeks and >24 weeks included total protein, albumin, globulin, calcium, and bilirubin (data not shown). Dramatic changes in bilirubin, GGT and BUN measured between younger and older age groups is potentially due to weaning, as breast milk is known to contain several growth factors and enzymes known to stimulate hepatocyte proliferation and liver function (45), which would alter as the animals reduce suckling behaviors. This also would explain why at the lower ages (40) these parameters shifted more in the MR animals compared to NR animals.

Diet appeared to also influence serum chemistries based on significant differences between breast-fed and formula-fed human infants. In one report, there were significantly higher levels in cholesterol, Trig, ALT, GGT, AST, and bilirubin in breast-fed human infants, whereas formula-fed human infants presented with significantly higher BUN and phosphate levels at both 4 and 8 weeks of age (45). Age-related variations also were noted in other species, and similar to comparably-aged piglets and humans (46, 47), rhesus infants presented with higher levels of iron (Figure 5A), phosphorous (Ph; Figure 5E), AlkP (Figure 5F), and GGT; Figure 5H), as well as glucose, AST and LDH (not shown) compared to adults. Additional serum biochemistry parameters influenced by nutrition source are calcium and phosphorus, and their metabolic pathways are intricate and directly related to renal physiology and bone growth (mineral deposition). Calcium and phosphorus are also affected by the increase in fetal calcium and phosphorus accumulation during *in utero* bone mineralization (active transplacental transport of Ca and P). This accumulation could possibly suppress fetal parathyroid hormone secretion and Vitamin D synthesis (and enhance fetal calcitonin secretion). In humans, the nadir for plasma calcium values is ~48 h post-partum. A study which compared breast-fed human infants aged 4 and 8 weeks old, reported significant differences for GGT, total protein, albumin, BUN, bilirubin and iron, indicating important changes in metabolic responses occurring at or soon after 4 weeks of age (45).

Specific subsets of immune cells are responsible for resistance to pathogens, stimulating tissue repair, and maintaining homeostasis. Humans *in utero* undergo distinct patterns in growth and immunological development compared to those after birth as occur in infants that are nursing, adolescents, adults and the elderly. While CBC analyses measure basic cell types based on nuclear morphology, cell size and granularity, cell staining with monoclonal antibodies, and subsequent flow cytometric analysis can provide a more specific breakdown of cellular subtypes.

Fetal immunity has been described as being mainly reliant on innate immune cells such as neutrophils, APCs, NK cells, and gamma-delta T cells, CD4+ T regulatory cells and Th17 cells (1, 48). During this stage of development, the fetal liver is responsible for hematopoiesis, but after birth this function transitions to the bone marrow (49). Newborns undergo rapid transformation in immune cell frequency and function, due to massive antigen exposure, changes in gut permeability, nutrition, tissue seeding of immune cells, maturation of secondary lymphoid organs, vaccination, and growth. Our results illustrate that lymphocyte numbers differ dramatically with age, and the developmental patterns are different between rhesus cohorts. We found that B cells, CD1c+ DCs, CM CD4+ T cells and CM CD8+ T cells, all increased drastically over the first 6 months of life but this was much more striking within both colony groups, when compared to research animals. This was in contrast to total and naïve T cell subsets, which decreased with age prominently in the research cohort. This has important consequences for the study of diseases that impact CD4 T cells counts, such as during HIV/SIV infection, and stresses the importance of using appropriate uninfected control animals.

Reports have shown that the human neonates <2 months of age have low numbers of NK cells, B cells, CD4+T cells, and CD8+ T cells that increase sharply and then plateau at ~4–6 months of age (50). After ~6 months of age, these cell populations slowly decline through adolescence (51). Neonates carry high numbers of monocytes, dendritic cells and neutrophils after birth that then decline over time (52). Overall, pediatric patients exhibit significantly higher numbers of CD4+T cells, CD8+ T cells, B cells (9), monocytes, dendritic cells (53), and neutrophils (1) compared to adults. An evaluation of Chinese-ancestry rhesus macaques aged 1, 6, and 11 years of age, demonstrated a negative correlation between age and lymphocyte subsets, with no differences in monocyte and dendritic cell populations over age (54). This report also described significant differences between males and females for all leukocytes studied except pDCs, with females having higher counts for T cell subsets, B cells, monocytes and myeloid DCs compared to males (54). Another study evaluated immune cells with age in Indian-ancestry rhesus macaques, and found significantly lower frequencies of monocytes and myeloid DCs in younger animals (age 1–4 years) compared to adult and elderly age groups (55). Stark declines in *percentage* of naïve T cells were apparent with increasing age, along with increased memory T cells, decline in CD4:CD8 ratio, and increase in frequency of CD8+ T cells. Another study similarly reported decreased B and T lymphocyte counts and CD4:CD8 ratios with increasing age, and also found that monocyte counts increased from infancy (0–48 weeks of age) to young adulthood (6–10 years old) (56). In yet another study about 29 nursery-reared rhesus macaques designated as neonates (0–4 weeks old), infants (6–22 weeks old), and juveniles (30–46 weeks old), all lymphocyte subsets increased dramatically from birth to 4 weeks of age and then stabilized throughout infancy into juvenile age groups, with declining CD4:CD8 ratios reflecting increase over time in CD8+ T cell populations (57). This report also noted that naïve T cells comprised the majority of T cell populations, were detected at high frequencies throughout all age groups, and thus did not significantly differ over time.

Additional publications have emphasized the importance of early rearing environments (58). Older adults separated from their mothers and caged in isolation for the first 9 months of life presented with higher mortality rates and lower CD4:CD8 ratios than adults that were mother-reared. This early life experience was also associated with higher NK cell frequency and immune function (58). Surprisingly, we reported few significant differences between Colony MR and Colony NR groups, compared to analyses between both NR cohorts. Despite identical diets and housing conditions, our NR animal cohorts exhibited markedly different immune cell frequencies with development. This may be related to Research NR animals undergoing more frequent anesthetic events and research-related procedures as well as underlying factors we could not control such as maternal health, and/or gestational age. A study by Lubach et al. (59) found that rearing condition impacted lymphocyte subsets such that NR rhesus macaques had significantly higher CD4+ to CD8+ ratios at 6, 12, 18, and 24 months resulting from lower CD8+ T cells. These studies also reported higher lymphoproliferative responses and lower NK cell activity in NR animals compared to the MR cohort.

Cytokines encompass a large array of soluble mediators secreted by activated immune cells that influence function and migration of various cell types. This diverse group of chemical communicators includes but is not limited to chemokines, growth factors, lymphokines, interleukins (ILs), interferons (IFNs), and tumor necrosis factors (TNFs). While their effects are not strictly dichotomous, they have been largely described as either proinflammatory or anti-inflammatory in nature and their relative levels in plasma can offer insight into a patient's general immune status, infection, growth, and hematopoiesis. Given that cytokines are commonly used as markers for immune correlates of systemic biological impact, critical fluctuations that occur during early development, age-specific reference ranges, and effects from environmental influences need to be further evaluated for more reliable interpretation of pediatric data.

The lower proinflammatory cytokines (IL-1β and IL12/23p40) and higher anti-inflammatory cytokines (IL-10 and IL-13) in plasma samples from our pediatric animals is consistent with previous reports that infants are skewed toward Th2 immunity and tolerance compared to adults. Interestingly the higher levels of TNFα, a pro-inflammatory cytokine, may in fact reflect pediatric reliance on innate immune responses by the major producers and targets of TNFα that include monocytes and macrophages (60). Additional notable differences between our pediatric animals and the adult animals include levels of G-CSF, IL-2, IL-4, and IL-5. Secretion of these cytokines in response to TLR ligands as surrogates for infectious agents, has also been reported to occur at significantly different levels from PBMCs harvested from infant (birth to 1 year of age), adolescent (3–4 years old), and young adult macaques (6–10 years old) (56). Of note, infant PBMCs produced significantly less IL-2, IL-4, IL-6, IL-17, IFNγ, IL-13, TNFα, and IL-10 compared to PBMCs from older age groups. Basal and induced cytokine levels thus appear highly age-dependent and clinical research reference ranges ideally should reflect this phenomenon. In a different study using young (1–4 years), adult (8–15 years), and aged (≥19 years) macaques, levels of the circulating cytokines, IL-6, IL-1ra, and IL-17 were higher in the aged group, while IL-8, IL12/23p40, and IL-15 were highest in the young animals (55). Of note, Research Colony animals exhibited higher levels of IL-2 (Figure 11A), IL-1β (Figure 11B), IL-6 (Figure 11E), and TNFα (Figure 11H) compared to both MR and NR colony animals at 12–16 weeks of age.

Shifts in cytokine levels over time also reflect age-related immunological development (27, 61). Historically, pediatric groups had been described as being biased for anti-inflammatory cytokines, while geriatric groups were skewed for pro-inflammatory cytokines in the plasma. Human fetuses and neonates also appear biased for anti-inflammatory cytokines and less developed adaptive immunity that contribute to maintenance of maternal-fetal tolerance during pregnancy. Limited data have been published on human neonatal plasma cytokine levels, primarily based on cord blood studies, that less accurately represent neonatal blood levels. Also, many of these studies did not report or classify maternal health nor stratified data by gestational age. The importance of gestational age was highlighted in a few studies demonstrating significant differences in cytokine levels between preterm and term neonates (61). For example, serum levels of 14 cytokines, including IL-6, IL-10, TNFα, IFNγ, and monocyte chemoattractant protein (MCP-1) differed between human infants born 30–32 weeks vs. after 36 weeks of gestation (62). However, these data were inconsistent with other studies reporting on cord blood cytokine levels after different gestational ages (63). A final caveat is that cytokines can be encapsulated in exosomes that if not properly lysed, may result in lower detection levels of cytokines thereby limiting data interpretation and reinforcing a need better standardization of blood processing methods for cytokine quantification (64).

Pediatric data often are analyzed using adult data or results from younger animals of wide age ranges as a reference or standard. Here, however, we have demonstrated that such reference comparisons can be misleading. Given the limited access to healthy human pediatric sampling, the pediatric rhesus macaque model has proven invaluable for helping translate what constitutes normal, healthy early development in a growing newborn. Immune cell ontogeny throughout fetal development and soon after birth appears extremely similar between rhesus macaques and humans. Furthermore, non-human primates exhibit rich social complexity and are susceptible to many related biological, behavioral, and psychological diseases as human children. In this study, the focus was on data collected from blood samples of rhesus macaques during the first year of life and raised under differing circumstances. The age-binning analysis applied in this study revealed that commonly used clinical measurements changed drastically and were not always comparable between age groups during the first year of life. Thus, it becomes important to carefully age match control groups in any biological investigations on very young animals. Many apparent developmental changes measured in our study were not always detected in reports on similar pediatric cohorts, with different trajectories over time and from different influences from of rearing on rate of immunological maturity. These differences between MR and NR reflect effects of passive immunity from breast milk, differing antigen exposures, and broader impacts of housing, husbandry, and experimental manipulation. Thus, the data presented here reinforce a need for clearer definition and description of ages of animals as well as environmental factors used to address pediatric development in clinical and experimental research. These data also provide a foundation to characterize immune responses and cell types within the tissues during developmental stages during and beyond the first year of life. The results of this study also are expected to improve standardization and interpretations of results from studies using pediatric rhesus macaques.

## Data Availability Statement

The raw data supporting the conclusions of this article will be made available by the authors, without undue reservation.

## Ethics Statement

The animal study was reviewed and approved by Tulane Institutional Animal Care & Use Committee.

## Author Contributions

KM, MK, and NS designed the experiments, analyzed and interpreted data, and wrote the manuscript. JT, KF, MG, and ED contributed data and participated in writing and editing the manuscript. AA organized the raw data for sharing purposes. All authors approved submission of the manuscript.

## Conflict of Interest

The authors declare that the research was conducted in the absence of any commercial or financial relationships that could be construed as a potential conflict of interest.
