# Supplementary material for: Clinical and Immunological Metrics During Pediatric Rhesus Macaque Development
Source: Front Pediatr. 2020 Jul 16;8:388. doi: 10.3389/fped.2020.00388 (PMC7378395; doi:10.3389/fped.2020.00388)
Supplement: Supplementary file 1 [file Table_1.docx]

**Table S1. Demographic information of pediatric rhesus macaques**

| **Animal group** | **Numbers of animals and samples** | | | |
| --- | --- | --- | --- | --- |
|  |  | **Females** | **Males** | **Total** |
| **Colony MR** | samples | 62 | 65 | 127 |
|  | animals | 56 | 61 | 117 |
| **Colony NR** | samples | 25 | 28 | 53 |
|  | animals | 9 | 12 | 21 |
| **Research NR** | samples | 61 | 102 | 163 |
|  | animals | 5 | 5 | 10 |
| **Research MR** | samples | 1 | 2 | 3 |
|  | animals | 1 | 2 | 3 |
| **All Colony** | samples | 87 | 93 | 180 |
|  | animals | 65 | 73 | 138 |
| **All MR** | samples | 63 | 67 | 130 |
|  | animals | 57 | 63 | 120 |
| **All NR** | samples | 86 | 130 | 216 |
|  | animals | 14 | 17 | 31 |
| **Total** | **samples** | **149** | **197** | **346** |
|  | **animals** | **71** | **80** | **151** |
