# Supplementary material for: Clinical and Immunological Metrics During Pediatric Rhesus Macaque Development
Source: Front Pediatr. 2020 Jul 16;8:388. doi: 10.3389/fped.2020.00388 (PMC7378395; doi:10.3389/fped.2020.00388)
Supplement: Supplementary file 2 [file Table_2.docx]

**Table S2. Immunophenotyping by Flow Cytometry**

| **Markers/gating** | **Cell type** |
| --- | --- |
| FSC-HxFSC-A single cells, SSCxFSC lymphocyte gate, CD3+ | Total T cells |
| FSC-HxFSC-A single cells, SSCxFSC lymphocyte gate, CD3+CD4+CD8- | CD4+ T cells |
| FSC-HxFSC-A single cells, SSCxFSC lymphocyte gate, CD3+ CD4+CD8-CD28+CD95- | Naïve CD4+ T cells |
| FSC-HxFSC-A single cells, SSCxFSC lymphocyte gate, CD3+ CD4+CD8-CD28+CD95+ | Central memory CD4+ T cells (CM) |
| FSC-HxFSC-A single cells, SSCxFSC lymphocyte gate, CD3+ CD4+CD8-CD28-CD95+ | Effector memory CD4+ T cells (EM) |
| FSC-HxFSC-A single cells, SSCxFSC lymphocyte gate, CD3+CD4-CD8+ | CD8+ T cells |
| FSC-HxFSC-A single cells, SSCxFSC lymphocyte gate, CD3+ CD4-CD8+CD28+CD95- | Naïve CD8+ T cells |
| FSC-HxFSC-A single cells, SSCxFSC lymphocyte gate, CD3+ CD4-CD8+CD28+CD95+ | Central memory CD8+ T cells (CM) |
| FSC-HxFSC-A single cells, SSCxFSC lymphocyte gate, CD3+ CD4-CD8+CD28-CD95+ | Effector memory CD8+ T cells (EM) |
| FSC-HxFSC-A single cells, HLA-DR+CD3-CD20+ | B cells |
| FSC-HxFSC-A single cells, HLA-DR+CD20-CD11b+CD14+ | Total monocytes |
| FSC-HxFSC-A single cells, HLA-DR+CD20-CD11b+CD14+CD16- | Classical monocytes |
| FSC-HxFSC-A single cells, HLA-DR+CD20-CD11b+CD14+CD16+ | Intermediate monocytes |
| FSC-HxFSC-A single cells, HLA-DR+CD20-CD11b+CD14-CD16+ | Non-classical monocytes |
| FSC-HxFSC-A single cells, HLA-DR+CD20-CD11b-CD16-CD1c+CD123- | CD1c+ dendritic cells |
| FSC-HxFSC-A single cells, HLA-DR+CD20-CD11b-CD16-CD1c-CD123+ | CD123+ plasmacytoid dendritic cells (pDCs) |
| FSC-HxFSC-A single cells, HLA-DR-CD3-CD8+ | Natural killer cells (NK) |
