# Supplementary material for: Clinical and Immunological Metrics During Pediatric Rhesus Macaque Development
Source: Front Pediatr. 2020 Jul 16;8:388. doi: 10.3389/fped.2020.00388 (PMC7378395; doi:10.3389/fped.2020.00388)
Supplement: Supplementary file 3 [file Table_3.docx]

**Table S3. Expanded Pediatric Complete Blood Counts and Serum Chemistry**

|  |  |  |  | **95% CI** | |  |  | **Number of Samples** |
| --- | --- | --- | --- | --- | --- | --- | --- | --- |
|  |  | **Mean** | **SD** | **Lower** | **Upper** | **Median** | **Range** |  |
| **% Neutrophils** |  |  |  |  |  |  |  |  |
|  | **All Colony** | **35.5** | **13.8** | **33.4** | **37.7** | **32.9** | **9.7 - 82.5** | **164** |
|  | Colony MR | 32.5 | 12.1 | 30.3 | 34.8 | 31.0 | 11.7 - 82.5 | 116 |
|  | Colony NR | 42.8 | 15.3 | 38.3 | 47.2 | 42.7 | 9.7 - 72.7 | 48 |
|  | Research NR | 30.0 | 12.5 | 28.0 | 32.0 | 29.0 | 7.3 - 78.9 | 152 |
| **% Lymphocytes** |  |  |  |  |  |  |  |  |
|  | **All Colony** | **58.0** | **13.5** | **55.9** | **60.0** | **59.6** | **13.8 - 86.3** | **164** |
|  | Colony MR | 60.3 | 12.1 | 58.1 | 62.6 | 60.8 | 13.8 - 84.8 | 116 |
|  | Colony NR | 52.3 | 14.9 | 47.9 | 56.6 | 51.5 | 24.6 - 86.3 | 48 |
|  | Research NR | 63.1 | 11.9 | 61.2 | 65.0 | 64.2 | 18.5 - 88.1 | 152 |
| **% Monocytes** |  |  |  |  |  |  |  |  |
|  | **All Colony** | **4.0** | **1.3** | **3.8** | **4.2** | **3.8** | **1 - 9** | **164** |
|  | Colony MR | 4.1 | 1.4 | 3.8 | 4.3 | 3.8 | 1 - 9 | 116 |
|  | Colony NR | 3.8 | 1.2 | 3.4 | 4.1 | 3.7 | 1.2 - 8.5 | 48 |
|  | Research NR | 5.3 | 1.9 | 5.0 | 5.6 | 5.2 | 1.3 - 15 | 152 |
| **% Eosinophils** |  |  |  |  |  |  |  |  |
|  | **All Colony** | **2.0** | **3.0** | **1.6** | **2.5** | **1.1** | **0 - 27** | **164** |
|  | Colony MR | 2.5 | 3.4 | 1.9 | 3.2 | 1.4 | 0 - 27 | 116 |
|  | Colony NR | 0.8 | 1.0 | 0.5 | 1.1 | 0.4 | 0.1 - 5.7 | 48 |
|  | Research NR | 1.2 | 1.2 | 1.0 | 1.4 | 0.8 | 0 - 7.3 | 152 |
| **% Basophils** |  |  |  |  |  |  |  |  |
|  | **All Colony** | **0.5** | **0.3** | **0.5** | **0.6** | **0.5** | **0 - 1.5** | **164** |
|  | Colony MR | 0.6 | 0.3 | 0.5 | 0.6 | 0.5 | 0 - 1.5 | 116 |
|  | Colony NR | 0.5 | 0.2 | 0.4 | 0.5 | 0.4 | 0.1 - 1.1 | 48 |
|  | Research NR | 0.5 | 0.3 | 0.5 | 0.6 | 0.5 | 0 - 1.8 | 152 |
| **# Neutrophils (x10e3/uL)** | |  |  |  |  |  |  |  |
|  | **All Colony** | **3.59** | **2.05** | **3.27** | **3.90** | **3.12** | **0.51 - 10.2** | **164** |
|  | Colony MR | 3.58 | 1.96 | 3.22 | 3.94 | 3.22 | 0.8 - 9.25 | 116 |
|  | Colony NR | 3.61 | 2.27 | 2.95 | 4.27 | 2.92 | 0.51 - 10.2 | 48 |
|  | Research NR | 2.30 | 1.29 | 2.10 | 2.51 | 2.07 | 0.21 - 8.36 | 152 |
| **# Lymphocytes (x10e3/uL)** | |  |  |  |  |  |  |  |
|  | **All Colony** | **5.67** | **2.70** | **5.25** | **6.08** | **4.97** | **1.21 - 17.2** | **164** |
|  | Colony MR | 6.41 | 2.84 | 5.89 | 6.94 | 6.12 | 1.21 - 17.2 | 116 |
|  | Colony NR | 3.86 | 0.95 | 3.58 | 4.13 | 3.90 | 1.7 - 5.95 | 48 |
|  | Research NR | 4.66 | 1.52 | 4.42 | 4.91 | 4.50 | 1.51 - 9.48 | 152 |
| **# Monocytes (x10e3/uL)** | |  |  |  |  |  |  |  |
|  | **All Colony** | **0.40** | **0.21** | **0.36** | **0.43** | **0.34** | **0.05 - 1.49** | **164** |
|  | Colony MR | 0.43 | 0.22 | 0.39 | 0.48 | 0.42 | 0.06 - 1.49 | 116 |
|  | Colony NR | 0.30 | 0.16 | 0.25 | 0.34 | 0.28 | 0.05 - 0.89 | 48 |
|  | Research NR | 0.38 | 0.15 | 0.35 | 0.40 | 0.35 | 0.13 - 0.92 | 152 |
| **# Eosinophils (x10e3/uL)** | |  |  |  |  |  |  |  |
|  | **All Colony** | **0.24** | **0.46** | **0.17** | **0.31** | **0.09** | **0 - 4.656** | **164** |
|  | Colony MR | 0.32 | 0.52 | 0.22 | 0.42 | 0.15 | 0 - 4.656 | 116 |
|  | Colony NR | 0.05 | 0.07 | 0.03 | 0.07 | 0.03 | 0.01 - 0.4 | 48 |
|  | Research NR | 0.09 | 0.10 | 0.07 | 0.10 | 0.05 | 0 - 0.51 | 152 |
| **# Basophils (x10e3/uL)** | |  |  |  |  |  |  |  |
|  | **All Colony** | **0.05** | **0.03** | **0.05** | **0.06** | **0.05** | **0 - 0.17** | **164** |
|  | Colony MR | 0.06 | 0.03 | 0.05 | 0.07 | 0.05 | 0 - 0.17 | 116 |
|  | Colony NR | 0.04 | 0.02 | 0.03 | 0.04 | 0.03 | 0.01 - 0.11 | 48 |
|  | Research NR | 0.04 | 0.02 | 0.04 | 0.04 | 0.04 | 0 - 0.17 | 152 |
| **WBC (x10e3/uL)** |  |  |  |  |  |  |  |  |
|  | **All Colony** | **10.03** | **4.22** | **9.38** | **10.69** | **9.21** | **3.73 - 29.48** | **164** |
|  | Colony MR | 10.94 | 4.43 | 10.12 | 11.75 | 10.19 | 3.73 - 29.48 | 116 |
|  | Colony NR | 7.85 | 2.62 | 7.09 | 8.62 | 7.52 | 3.74 - 14.94 | 48 |
|  | Research NR | 7.47 | 2.09 | 7.13 | 7.80 | 7.35 | 2.41 - 13.59 | 152 |
| **RBC (x10e6/uL)** |  |  |  |  |  |  |  |  |
|  | **All Colony** | **5.71** | **0.50** | **5.63** | **5.78** | **5.68** | **4.58 - 7.4** | **164** |
|  | Colony MR | 5.75 | 0.54 | 5.65 | 5.84 | 5.73 | 4.58 - 7.4 | 116 |
|  | Colony NR | 5.62 | 0.37 | 5.51 | 5.72 | 5.60 | 4.88 - 6.56 | 48 |
|  | Research NR | 5.37 | 0.41 | 5.31 | 5.44 | 5.40 | 4.31 - 6.84 | 152 |
| **Hemoglobin (g/dL)** |  |  |  |  |  |  |  |  |
|  | **All Colony** | **12.1** | **1.4** | **11.9** | **12.4** | **12.3** | **6.3 - 17.8** | **164** |
|  | Colony MR | 12.1 | 1.6 | 11.8 | 12.3 | 12.2 | 6.3 - 17.8 | 116 |
|  | Colony NR | 12.4 | 0.6 | 12.2 | 12.6 | 12.4 | 10.7 - 13.8 | 48 |
|  | Research NR | 12.6 | 1.2 | 12.4 | 12.7 | 12.4 | 10.9 - 19 | 152 |
| **Hematocrit (%)** |  |  |  |  |  |  |  |  |
|  | **All Colony** | **38.3** | **3.4** | **37.7** | **38.8** | **38.7** | **24.1 - 52.8** | **164** |
|  | Colony MR | 38.0 | 3.9 | 37.3 | 38.7 | 38.4 | 24.1 - 52.8 | 116 |
|  | Colony NR | 38.9 | 1.6 | 38.5 | 39.4 | 39.0 | 34.2 - 42.8 | 48 |
|  | Research NR | 39.2 | 3.1 | 38.7 | 39.7 | 38.9 | 34.2 - 56.8 | 152 |
| **MCV (fL)** |  |  |  |  |  |  |  |  |
|  | **All Colony** | **67.3** | **6.5** | **66.3** | **68.3** | **68.3** | **39.8 - 83.8** | **164** |
|  | Colony MR | 66.4 | 7.3 | 65.1 | 67.8 | 67.7 | 39.8 - 83.8 | 116 |
|  | Colony NR | 69.5 | 3.3 | 68.5 | 70.4 | 69.6 | 61.9 - 77.1 | 48 |
|  | Research NR | 73.2 | 4.6 | 72.4 | 73.9 | 71.7 | 66.9 - 90.1 | 152 |
| **MCH (pg)** |  |  |  |  |  |  |  |  |
|  | **All Colony** | **21.4** | **2.5** | **21.0** | **21.8** | **21.7** | **10.4 - 27.8** | **164** |
|  | Colony MR | 21.1 | 2.9 | 20.6 | 21.6 | 21.2 | 10.4 - 27.8 | 116 |
|  | Colony NR | 22.1 | 1.0 | 21.8 | 22.4 | 22.3 | 19.6 - 24 | 48 |
|  | Research NR | 23.4 | 1.7 | 23.1 | 23.7 | 22.8 | 21.2 - 29.6 | 152 |
| **MCHC (g/dL)** |  |  |  |  |  |  |  |  |
|  | **All Colony** | **31.7** | **1.3** | **31.5** | **31.9** | **31.7** | **25.2 - 34.7** | **164** |
|  | Colony MR | 31.7 | 1.5 | 31.4 | 31.9 | 31.6 | 25.2 - 34.7 | 116 |
|  | Colony NR | 31.8 | 0.6 | 31.6 | 32.0 | 31.8 | 30.2 - 33.2 | 48 |
|  | Research NR | 32.0 | 0.8 | 31.8 | 32.1 | 31.9 | 30.1 - 34.8 | 152 |
| **RDW (%)** |  |  |  |  |  |  |  |  |
|  | **All Colony** | **14.8** | **3.4** | **14.3** | **15.3** | **13.9** | **11.7 - 35.7** | **163** |
|  | Colony MR | 15.4 | 3.8 | 14.7 | 16.1 | 14.1 | 11.7 - 35.7 | 115 |
|  | Colony NR | 13.4 | 0.7 | 13.2 | 13.6 | 13.4 | 12 - 15.6 | 48 |
|  | Research NR | 13.0 | 0.6 | 12.9 | 13.1 | 13.0 | 11.5 - 14.3 | 152 |
| **Platelets (x10e3/uL)** | |  |  |  |  |  |  |  |
|  | **All Colony** | **481.4** | **158.6** | **457.0** | **505.9** | **463.0** | **42-974** | **164** |
|  | Colony MR | 514.5 | 168.0 | 483.6 | 545.4 | 497.0 | 42-974 | 116 |
|  | Colony NR | 401.4 | 94.3 | 374.0 | 428.7 | 389.5 | 236-659 | 48 |
|  | Research NR | 474.7 | 128.1 | 454.2 | 495.3 | 461.5 | 227-994 | 152 |
| **MPV (fL)** |  |  |  |  |  |  |  |  |
|  | **All Colony** | **11.6** | **1.0** | **11.4** | **11.8** | **11.7** | **9.8 - 13.7** | **133** |
|  | Colony MR | 11.4 | 1.0 | 11.2 | 11.6 | 11.5 | 9.8 - 13.7 | 94 |
|  | Colony NR | 12.1 | 0.9 | 11.8 | 12.4 | 12.2 | 10.1 - 13.7 | 39 |
|  | Research NR | 11.9 | 1.1 | 11.7 | 12.1 | 11.9 | 10 - 14.4 | 125 |
| **Sodium (mEq/L)** |  |  |  |  |  |  |  |  |
|  | **All Colony** | **144** | **3** | **144** | **145** | **144** | **135 - 152** | **176** |
|  | Colony MR | 144 | 3 | 144 | 144 | 144 | 135 - 152 | 123 |
|  | Colony NR | 145 | 2 | 144 | 145 | 145 | 138 - 149 | 53 |
|  | Research NR | 144 | 2 | 144 | 144 | 144 | 140 - 149 | 139 |
| **Potassium (mEq/L)** |  |  |  |  |  |  |  |  |
|  | **All Colony** | **4.3** | **1.0** | **4.1** | **4.4** | **4.0** | **3.2 - 11.3** | **176** |
|  | Colony MR | 4.3 | 1.0 | 4.1 | 4.5 | 4.0 | 3.2 - 11.3 | 123 |
|  | Colony NR | 4.3 | 1.1 | 4.0 | 4.6 | 4.0 | 3.4 - 10.5 | 53 |
|  | Research NR | 4.5 | 0.9 | 4.3 | 4.6 | 4.3 | 3.1 - 8.5 | 139 |
| **Chloride (mEq/L** |  |  |  |  |  |  |  |  |
|  | **All Colony** | **108** | **2** | **108** | **109** | **108** | **98 - 113** | **176** |
|  | Colony MR | 108 | 2 | 108 | 108 | 108 | 98 - 112 | 123 |
|  | Colony NR | 109 | 3 | 108 | 110 | 109 | 99 - 113 | 53 |
|  | Research NR | 109 | 2 | 109 | 109 | 109 | 104 - 115 | 139 |
| **Total Protein (g/dL)** | |  |  |  |  |  |  |  |
|  | **All Colony** | **6.2** | **0.5** | **6.2** | **6.3** | **6.3** | **4.8 - 7.5** | **175** |
|  | Colony MR | 6.2 | 0.6 | 6.1 | 6.3 | 6.3 | 4.8 - 7.5 | 122 |
|  | Colony NR | 6.3 | 0.4 | 6.2 | 6.4 | 6.4 | 5.4 - 7.2 | 53 |
|  | Research NR | 5.9 | 0.5 | 5.8 | 6.0 | 5.9 | 4.7 - 7.1 | 139 |
| **Albumin (g/dL)** |  |  |  |  |  |  |  |  |
|  | **All Colony** | **4.0** | **0.3** | **4.0** | **4.1** | **4.1** | **2.9 - 5.4** | **176** |
|  | Colony MR | 4.0 | 0.3 | 3.9 | 4.0 | 4.0 | 2.9 - 5.4 | 123 |
|  | Colony NR | 4.2 | 0.3 | 4.1 | 4.3 | 4.3 | 3.4 - 4.9 | 53 |
|  | Research NR | 3.8 | 0.4 | 3.8 | 3.9 | 3.9 | 2.7 - 4.5 | 139 |
| **Globulin (g/dL)** |  |  |  |  |  |  |  |  |
|  | **All Colony** | **2.2** | **0.4** | **2.1** | **2.3** | **2.1** | **0.6 - 3.9** | **175** |
|  | Colony MR | 2.3 | 0.5 | 2.2 | 2.3 | 2.3 | 0.6 - 3.9 | 122 |
|  | Colony NR | 2.1 | 0.2 | 2.0 | 2.1 | 2.0 | 1.8 - 2.8 | 53 |
|  | Research NR | 2.1 | 0.3 | 2.0 | 2.1 | 2.1 | 1 - 3.3 | 139 |
| **A/G Ratio** |  |  |  |  |  |  |  |  |
|  | **All Colony** | **1.9** | **0.5** | **1.8** | **2.0** | **1.9** | **0.9 - 7.2** | **175** |
|  | Colony MR | 1.9 | 0.6 | 1.7 | 2.0 | 1.8 | 0.9 - 7.2 | 122 |
|  | Colony NR | 2.1 | 0.2 | 2.0 | 2.1 | 2.1 | 1.4 - 2.6 | 53 |
|  | Research NR | 1.9 | 0.4 | 1.8 | 2.0 | 1.9 | 1 - 4.2 | 139 |
| **Calcium (mg/dL)** |  |  |  |  |  |  |  |  |
|  | **All Colony** | **10.1** | **0.5** | **10.0** | **10.2** | **10.1** | **8.4 - 11.8** | **172** |
|  | Colony MR | 10.2 | 0.5 | 10.1 | 10.3 | 10.2 | 9.1 - 11.8 | 119 |
|  | Colony NR | 9.9 | 0.4 | 9.8 | 10.0 | 9.9 | 8.4 - 10.8 | 53 |
|  | Research NR | 10.0 | 0.4 | 9.9 | 10.1 | 9.9 | 9 - 11.5 | 138 |
| **Total Bilirubin (mg/dL)** | |  |  |  |  |  |  |  |
|  | **All Colony** | **0.17** | **0.11** | **0.15** | **0.19** | **0.14** | **0.04 - 0.96** | **174** |
|  | Colony MR | 0.17 | 0.11 | 0.15 | 0.19 | 0.14 | 0.04 - 0.96 | 121 |
|  | Colony NR | 0.18 | 0.11 | 0.15 | 0.21 | 0.15 | 0.04 - 0.84 | 53 |
|  | Research NR | 0.16 | 0.08 | 0.15 | 0.18 | 0.14 | 0.02 - 0.56 | 138 |
| **BUN (mg/dL)** |  |  |  |  |  |  |  |  |
|  | **All Colony** | **15.9** | **5.3** | **15.1** | **16.7** | **15.0** | **6 - 31** | **176** |
|  | Colony MR | 15.0 | 4.9 | 14.2 | 15.9 | 14.0 | 6 - 31 | 123 |
|  | Colony NR | 17.8 | 5.9 | 16.2 | 19.4 | 19.0 | 9 - 28 | 53 |
|  | Research NR | 12.9 | 6.1 | 11.9 | 13.9 | 11.0 | 4 - 28 | 139 |
| **Glucose (mg/dL)** |  |  |  |  |  |  |  |  |
|  | **All Colony** | **83** | **31** | **78** | **87** | **75** | **31 - 180** | **176** |
|  | Colony MR | 91 | 33 | 85 | 96 | 85 | 31 - 180 | 123 |
|  | Colony NR | 64 | 14 | 60 | 68 | 67 | 31 - 98 | 53 |
|  | Research NR | 76 | 27 | 72 | 81 | 70 | 24 - 192 | 139 |
| **Creatinine (mg/dL)** |  |  |  |  |  |  |  |  |
|  | **All Colony** | **0.37** | **0.11** | **0.35** | **0.38** | **0.34** | **0.18 - 0.78** | **176** |
|  | Colony MR | 0.40 | 0.11 | 0.38 | 0.42 | 0.37 | 0.21 - 0.78 | 123 |
|  | Colony NR | 0.30 | 0.06 | 0.28 | 0.32 | 0.29 | 0.18 - 0.47 | 53 |
|  | Research NR | 0.28 | 0.04 | 0.27 | 0.29 | 0.28 | 0.12 - 0.37 | 139 |
| **Phosphorus (mg/dL)** | |  |  |  |  |  |  |  |
|  | **All Colony** | **7.4** | **1.0** | **7.2** | **7.5** | **7.3** | **4.8 - 10.5** | **173** |
|  | Colony MR | 7.3 | 1.0 | 7.1 | 7.4 | 7.2 | 4.8 - 10.5 | 120 |
|  | Colony NR | 7.5 | 0.9 | 7.3 | 7.8 | 7.4 | 5.5 - 10.1 | 53 |
|  | Research NR | 8.4 | 1.1 | 8.2 | 8.6 | 8.4 | 5.7 - 11.1 | 138 |
| **Iron (ug/dL)** |  |  |  |  |  |  |  |  |
|  | **All Colony** | **118** | **55** | **110** | **126** | **115** | **12 - 285** | **174** |
|  | Colony MR | 116 | 57 | 105 | 126 | 119 | 12 - 279 | 121 |
|  | Colony NR | 124 | 51 | 110 | 138 | 113 | 51 - 285 | 53 |
|  | Research NR | 157 | 53 | 149 | 166 | 154 | 46 - 314 | 139 |
| **Alkaline Phosphatase (mg/dL)** | |  |  |  |  |  |  |  |
|  | **All Colony** | **803** | **282** | **761** | **845** | **742** | **370 - 1809** | **174** |
|  | Colony MR | 807 | 294 | 754 | 859 | 768 | 370 - 1809 | 121 |
|  | Colony NR | 796 | 256 | 725 | 866 | 695 | 457 - 1608 | 53 |
|  | Research NR | 946 | 276 | 899 | 992 | 900 | 481 - 1680 | 138 |
| **AST (U/L)** |  |  |  |  |  |  |  |  |
|  | **All Colony** | **62** | **60** | **53** | **70** | **45** | **23 - 611** | **176** |
|  | Colony MR | 57 | 34 | 51 | 63 | 45 | 26 - 223 | 123 |
|  | Colony NR | 73 | 95 | 47 | 99 | 44 | 23 - 611 | 53 |
|  | Research NR | 66 | 38 | 60 | 73 | 55 | 30 - 269 | 139 |
| **ALT (U/L)** |  |  |  |  |  |  |  |  |
|  | **All Colony** | **28** | **13** | **26** | **30** | **26** | **2 - 84** | **176** |
|  | Colony MR | 26 | 13 | 24 | 28 | 23 | 2 - 84 | 123 |
|  | Colony NR | 34 | 12 | 31 | 38 | 33 | 14 - 83 | 53 |
|  | Research NR | 36 | 13 | 34 | 38 | 33 | 17 - 90 | 139 |
| **LDH (U/L)** |  |  |  |  |  |  |  |  |
|  | **All Colony** | **816** | **465** | **747** | **886** | **696** | **134 - 3512** | **173** |
|  | Colony MR | 858 | 458 | 776 | 941 | 735 | 296 - 3512 | 121 |
|  | Colony NR | 719 | 471 | 588 | 850 | 621 | 134 - 2798 | 52 |
|  | Research NR | 796 | 443 | 722 | 870 | 671 | 28 - 2642 | 139 |
| **Cholesterol (mg/dL)** | |  |  |  |  |  |  |  |
|  | **All Colony** | **159** | **45** | **153** | **166** | **152** | **71 - 406** | **174** |
|  | Colony MR | 167 | 49 | 158 | 176 | 159 | 71 - 406 | 121 |
|  | Colony NR | 142 | 25 | 135 | 149 | 144 | 80 - 189 | 53 |
|  | Research NR | 138 | 22 | 134 | 142 | 138 | 88 - 205 | 139 |
| **BUN/Creat Ratio** |  |  |  |  |  |  |  |  |
|  | **All Colony** | **46.7** | **20.7** | **43.6** | **49.7** | **42.0** | **16.7 - 100** | **176** |
|  | Colony MR | 41.0 | 18.7 | 37.6 | 44.3 | 36.4 | 16.7 - 100 | 123 |
|  | Colony NR | 59.9 | 19.0 | 54.7 | 65.2 | 61.8 | 31.7 - 100 | 53 |
|  | Research NR | 45.9 | 18.7 | 42.7 | 49.0 | 40.0 | 16.7 - 90 | 139 |
| **Triglycerides (mg/dL)** | |  |  |  |  |  |  |  |
|  | **All Colony** | **57** | **23** | **54** | **60** | **51** | **25 - 175** | **173** |
|  | Colony MR | 57 | 25 | 52 | 61 | 50 | 25 - 175 | 120 |
|  | Colony NR | 58 | 17 | 53 | 63 | 56 | 29 - 107 | 53 |
|  | Research NR | 52 | 39 | 45 | 59 | 45 | 12 - 406 | 138 |
| **GGT (U/L)** |  |  |  |  |  |  |  |  |
|  | **All Colony** | **91** | **31** | **86** | **96** | **85** | **29 - 226** | **173** |
|  | Colony MR | 91 | 35 | 85 | 98 | 85 | 29 - 226 | 121 |
|  | Colony NR | 90 | 21 | 84 | 96 | 85 | 54 - 157 | 52 |
|  | Research NR | 90 | 24 | 86 | 94 | 88 | 47 - 159 | 138 |
