# Supplementary material for: Clinical and Immunological Metrics During Pediatric Rhesus Macaque Development
Source: Front Pediatr. 2020 Jul 16;8:388. doi: 10.3389/fped.2020.00388 (PMC7378395; doi:10.3389/fped.2020.00388)
Supplement: Supplementary file 4 [file Table_4.docx]

**Table S4. Expanded Pediatric Peripheral Immunophenotyping**

|  |  |  |  | **95% CI** | |  |  | **Number of Samples** |
| --- | --- | --- | --- | --- | --- | --- | --- | --- |
|  |  | **Mean** | **SD** | **Lower** | **Upper** | **Median** | **Range** |  |
| **CD3+ T cells (#/uL)** | |  |  |  |  |  |  |  |
|  | **All Colony** | **3819** | **2019** | **3506** | **4132** | **3452** | **784.7-10865** | **162** |
|  | Colony MR | 3777 | 2029 | 3400 | 4153 | 3384 | 815-10283 | 114 |
|  | Colony NR | 3920 | 2013 | 3336 | 4505 | 3564 | 784.7-10865 | 48 |
|  | Research NR | 2964 | 1263 | 2638 | 3291 | 2880 | 840.3-7152 | 60 |
| **CD20+HLA-DR+ B cells (#/uL)** | | |  |  |  |  |  |  |
|  | **All Colony** | **2385** | **1477** | **2156** | **2614** | **2126** | **107-8404** | **162** |
|  | Colony MR | 2467 | 1518 | 2186 | 2749 | 2168 | 107-8404 | 114 |
|  | Colony NR | 2189 | 1370 | 1791 | 2587 | 1840 | 240.9-6641 | 48 |
|  | Research NR | 1339 | 717.8 | 1154 | 1524 | 1195 | 63.87-3640 | 60 |
| **CD8+CD3- NK cells (#/uL)** | |  |  |  |  |  |  |  |
|  | **All Colony** | **433.9** | **521.8** | **352.9** | **514.8** | **272.3** | **0.12-3790** | **162** |
|  | Colony MR | 467.6 | 590.6 | 358 | 577.1 | 272.3 | 0.12-3790 | 114 |
|  | Colony NR | 353.9 | 291.7 | 269.2 | 438.5 | 271.3 | 9.879-1492 | 48 |
|  | Research NR | 251.6 | 181 | 204.8 | 298.3 | 192.7 | 24.89-810.4 | 60 |
| **CD4+CD3+ T cells (#/uL)** | |  |  |  |  |  |  |  |
|  | **All Colony** | **2327** | **1240** | **2134** | **2519** | **2079** | **447.5-7410** | **162** |
|  | Colony MR | 2224 | 1167 | 2008 | 2441 | 1962 | 447.5-6040 | 114 |
|  | Colony NR | 2570 | 1381 | 2169 | 2971 | 2295 | 464.5-7410 | 48 |
|  | Research NR | 1953 | 920.2 | 1715 | 2190 | 1919 | 617.6-5714 | 60 |
| **CD14+CD11b+HLA-DR+ Monocytes (#/uL)** | | |  |  |  |  |  |  |
|  | **All Colony** | **472.5** | **465.5** | **400.3** | **544.7** | **317.6** | **38.47-3556** | **162** |
|  | Colony MR | 450 | 453.3 | 365.9 | 534.2 | 310.5 | 70.54-3556 | 114 |
|  | Colony NR | 525.9 | 494 | 382.4 | 669.4 | 353 | 38.47-2728 | 48 |
|  | Research NR | 371.9 | 155.4 | 331.8 | 412 | 335.5 | 163.6-841.9 | 60 |
| **CD14-CD16+CD11b+HLA-DR+ Non-classical Monocytes (#/uL)** | | | | |  |  |  |  |
|  | **All Colony** | **118.5** | **123** | **99.46** | **137.6** | **81.11** | **0.93-786.9** | **162** |
|  | Colony MR | 104 | 105.8 | 84.42 | 123.7 | 73.04 | 3.07-591.8 | 114 |
|  | Colony NR | 153 | 152.2 | 108.8 | 197.2 | 127.6 | 0.93-786.9 | 48 |
|  | Research NR | 57.3 | 44.87 | 45.71 | 68.89 | 48.22 | 1.37-173.1 | 60 |
| **CD14+CD11b+CD16-HLA-DR+ Classical Monocytes (#/uL)** | | | | |  |  |  |  |
|  | **All Colony** | **406** | **418.4** | **341.1** | **470.9** | **272.5** | **36.53-3399** | **162** |
|  | Colony MR | 385.3 | 412.3 | 308.8 | 461.8 | 268.3 | 63.81-3399 | 114 |
|  | Colony NR | 455.1 | 433 | 329.4 | 580.9 | 317.7 | 36.53-2394 | 48 |
|  | Research NR | 336 | 142.1 | 299.2 | 372.7 | 311.4 | 141.5-824.7 | 60 |
| **CD1c+HLA-DR+ DCs (#/uL)** | |  |  |  |  |  |  |  |
|  | **All Colony** | **25.94** | **28.28** | **21.55** | **30.33** | **16.94** | **0.4044-242** | **162** |
|  | Colony MR | 25.75 | 29.03 | 20.37 | 31.14 | 18.42 | 0.54-242 | 114 |
|  | Colony NR | 26.38 | 26.69 | 18.63 | 34.13 | 13.9 | 0.4044-93.05 | 48 |
|  | Research NR | 15.6 | 11.19 | 12.71 | 18.49 | 12.59 | 1.98-70.74 | 60 |
| **CD123+HLA-DR+ DCs (#/uL)** | |  |  |  |  |  |  |  |
|  | **All Colony** | **8.049** | **10.79** | **6.375** | **9.723** | **5.41** | **0.2171-113.1** | **162** |
|  | Colony MR | 7.088 | 11.49 | 4.955 | 9.22 | 4.615 | 0.34-113.1 | 114 |
|  | Colony NR | 10.33 | 8.579 | 7.842 | 12.82 | 7.373 | 0.2171-40.32 | 48 |
|  | Research NR | 5.124 | 4.56 | 3.946 | 6.302 | 3.966 | 0-29.49 | 60 |
| **CD28+CD95-CD4+CD3+ Naïve T cells (#/uL)** |  |  |  |  |  |  |  |  |
|  | **All Colony** | **1771** | **1039** | **1609** | **1932** | **1596** | **47.85-6226** | **162** |
|  | Colony MR | 1657 | 993.1 | 1473 | 1841 | 1460 | 47.85-5070 | 114 |
|  | Colony NR | 2041 | 1106 | 1720 | 2362 | 1765 | 424-6226 | 48 |
|  | Research NR | 1650 | 868.2 | 1425 | 1874 | 1554 | 443.7-5266 | 60 |
| **CD28+CD95+CD4+CD3+ CM T cells (#/uL)** |  |  |  |  |  |  |  |  |
|  | **All Colony** | **520.3** | **330.6** | **469** | **571.6** | **442** | **33.07-1753** | **162** |
|  | Colony MR | 526.4 | 334.8 | 464.3 | 588.5 | 466.1 | 33.07-1753 | 114 |
|  | Colony NR | 505.9 | 323.5 | 411.9 | 599.8 | 424.7 | 38.5-1725 | 48 |
|  | Research NR | 284.4 | 93.99 | 260.1 | 308.7 | 299.5 | 49.65-469.6 | 60 |
| **CD28-CD95+CD4+CD3+ EM T cells (#/uL)** |  |  |  |  |  |  |  |  |
|  | **All Colony** | **16.78** | **33.09** | **11.65** | **21.92** | **5.475** | **0-251.5** | **162** |
|  | Colony MR | 22.07 | 38.18 | 14.98 | 29.15 | 7.68 | 0-251.5 | 114 |
|  | Colony NR | 4.232 | 4.062 | 3.053 | 5.411 | 2.833 | 0.2222-18.17 | 48 |
|  | Research NR | 7.689 | 14.98 | 3.819 | 11.56 | 2.19 | 0-75.58 | 60 |
| **CD3+ CD8+ T cells (#/uL)** |  |  |  |  |  |  |  |  |
|  | **All Colony** | **1194** | **787.5** | **1071** | **1316** | **971.7** | **156.7-5731** | **162** |
|  | Colony MR | 1237 | 863.8 | 1077 | 1397 | 1001 | 156.7-5731 | 114 |
|  | Colony NR | 1090 | 561.4 | 927.2 | 1253 | 926.8 | 278.9-2784 | 48 |
|  | Research NR | 868.8 | 531.6 | 731.5 | 1006 | 772.5 | 196.3-2614 | 60 |
| **CD28+CD95-CD8+CD3+ Naïve T cells (#/uL)** |  |  |  |  |  |  |  |  |
|  | **All Colony** | **633.6** | **360.8** | **577.6** | **689.6** | **557.5** | **87.73-1912** | **162** |
|  | Colony MR | 590 | 347.4 | 525.5 | 654.5 | 533 | 87.73-1758 | 114 |
|  | Colony NR | 737.2 | 374.4 | 628.5 | 845.9 | 647.9 | 200.4-1912 | 48 |
|  | Research NR | 556.1 | 267.8 | 486.9 | 625.3 | 533.1 | 155.7-1329 | 60 |
| **CD28+CD95+CD8+CD3+ CM T cells (#/uL)** |  |  |  |  |  |  |  |  |
|  | **All Colony** | **184.3** | **136.5** | **163.2** | **205.5** | **150.5** | **9.806-847.5** | **162** |
|  | Colony MR | 198.3 | 149.3 | 170.6 | 226 | 164.4 | 12.99-847.5 | 114 |
|  | Colony NR | 151.1 | 92.72 | 124.2 | 178 | 125.2 | 9.806-418.7 | 48 |
|  | Research NR | 90.56 | 44.65 | 79.03 | 102.1 | 82.22 | 9.33-182 | 60 |
| **CD28-CD95+CD8+CD3+ EM T cells (#/uL)** |  |  |  |  |  |  |  |  |
|  | **All Colony** | **366.8** | **462.1** | **295.1** | **438.5** | **204.6** | **8.673-3865** | **162** |
|  | Colony MR | 439.9 | 525.1 | 342.4 | 537.3 | 296.4 | 21.7-3865 | 114 |
|  | Colony NR | 193.2 | 157.3 | 147.5 | 238.9 | 149.2 | 8.673-847.5 | 48 |
|  | Research NR | 215.9 | 300.7 | 138.2 | 293.6 | 91.21 | 8.15-1482 | 60 |
| **%CD4 (of CD3)** |  |  |  |  |  |  |  |  |
|  | **All Colony** | **61.74** | **9.379** | **60.28** | **63.19** | **63.05** | **31-83.2** | **162** |
|  | Colony MR | 60.2 | 10.19 | 58.31 | 62.09 | 61.55 | 31-83.2 | 114 |
|  | Colony NR | 65.39 | 5.68 | 63.74 | 67.03 | 65.85 | 52.2-75.9 | 48 |
|  | Research NR | 66.47 | 11.03 | 63.62 | 69.32 | 69.1 | 41.7-86.4 | 60 |
| **%CD8 (of CD3)** |  |  |  |  |  |  |  |  |
|  | **All Colony** | **30.66** | **8.451** | **29.35** | **31.98** | **29.13** | **11.92-62.94** | **162** |
|  | Colony MR | 31.7 | 9.364 | 29.96 | 33.44 | 30.45 | 11.92-62.94 | 114 |
|  | Colony NR | 28.21 | 5.004 | 26.75 | 29.66 | 27.44 | 17.57-40.68 | 48 |
|  | Research NR | 28.8 | 9.649 | 26.31 | 31.29 | 27.56 | 11.63-52.13 | 60 |
| **CD4:CD8 Ratio** |  |  |  |  |  |  |  |  |
|  | **All Colony** | **2.253** | **0.9494** | **2.106** | **2.401** | **2.154** | **0.4925-6.284** | **162** |
|  | Colony MR | 2.181 | 1.048 | 1.987 | 2.375 | 2.06 | 0.4925-6.284 | 114 |
|  | Colony NR | 2.425 | 0.6365 | 2.24 | 2.61 | 2.401 | 1.291-4.321 | 48 |
|  | Research NR | 2.75 | 1.457 | 2.373 | 3.126 | 2.533 | 0.7999-7.43 | 60 |
